# Six Common Herbs with Distinctive Bioactive, Antioxidant Components. A Review of Their Separation Techniques

**DOI:** 10.3390/molecules26102920

**Published:** 2021-05-14

**Authors:** Antigoni Oreopoulou, Evanthia Choulitoudi, Dimitrios Tsimogiannis, Vassiliki Oreopoulou

**Affiliations:** 1 Laboratory of Food Chemistry and Technology, School of Chemical Engineering, National Technical University of Athens, 5 Iroon Polytechniou, 15780 Athens, Greece; antigoni@vioryl.gr (A.O.); echoulit@chemeng.ntua.gr (E.C.); ditsimog@chemeng.ntua.gr (D.T.); 2 Vioryl, Agricultural and Chemical Industry, Research S.A., 28th km National Road Athens-Lamia, 19014 Attiki, Greece; 3 NFA (Natural Food Additives), Laboratory of Natural Extracts Development, 6 Dios st, 17778 Athens, Greece

**Keywords:** rosemary, oregano, pink savory, lemon balm, St. John’s wort, saffron, extraction

## Abstract

Rosemary, oregano, pink savory, lemon balm, St. John’s wort, and saffron are common herbs wildly grown and easily cultivated in many countries. All of them are rich in antioxidant compounds that exhibit several biological and health activities. They are commercialized as spices, traditional medicines, or raw materials for the production of essential oils. The whole herbs or the residues of their current use are potential sources for the recovery of natural antioxidant extracts. Finding effective and feasible extraction and purification methods is a major challenge for the industrial production of natural antioxidant extracts. In this respect, the present paper is an extensive literature review of the solvents and extraction methods that have been tested on these herbs. Green solvents and novel extraction methods that can be easily scaled up for industrial application are critically discussed.

## 1. Introduction

During the last decades research has focused on natural antioxidants for use in food and cosmetics because they are more appealing to consumers. Plant material have been mostly investigated as potent sources, including cereals, seeds, spices, herbs, and some agroindustrial byproducts [1]. Among them, a considerable body of research efforts has been devoted to aromatic or medicinal plants, as they have been traditionally used for culinary purposes as spices or infusions, and also for their curative properties against mild disorders. Several aromatic and medicinal plants grow wildly in the Mediterranean region and in several other regions, while they are also cultivated.

**Figure 1:**
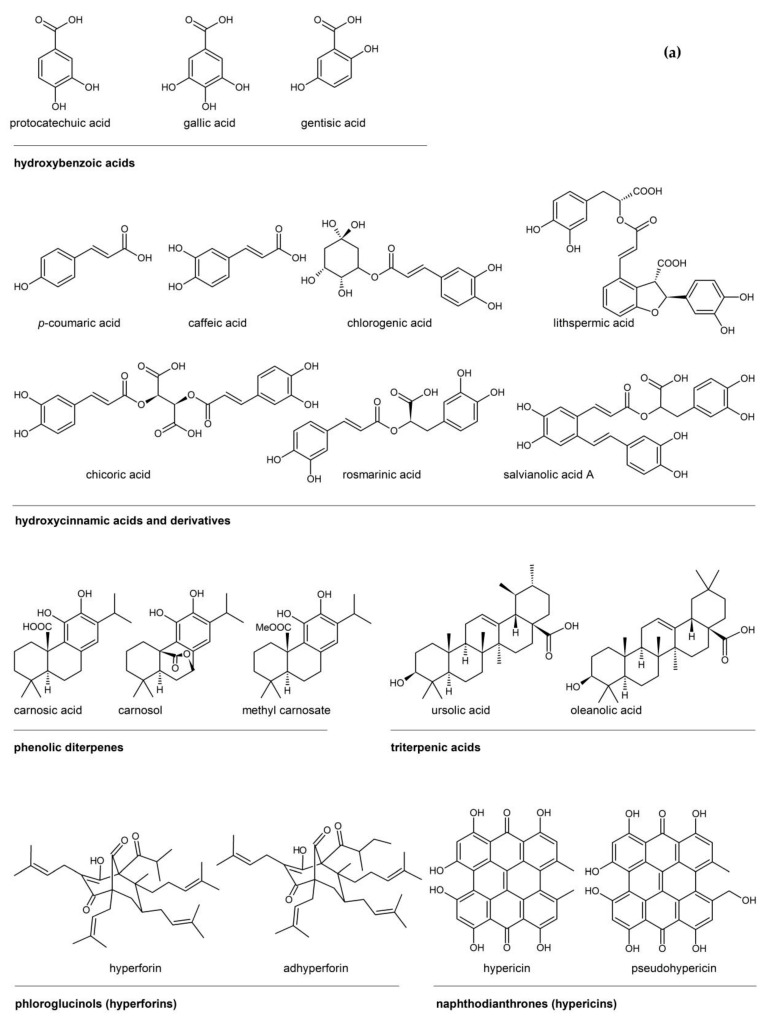

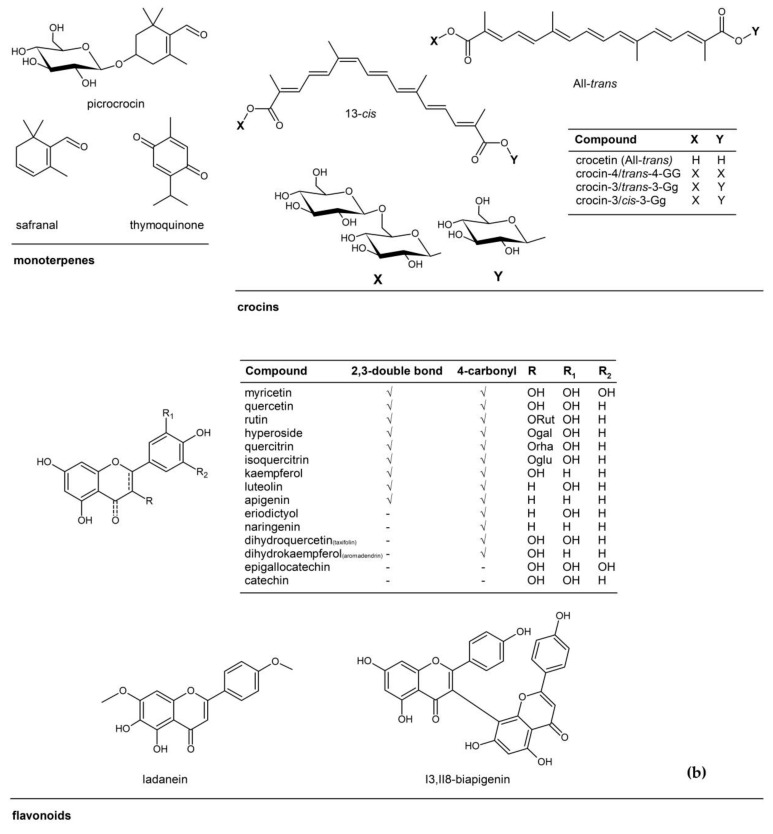
The structures of representative members from different groups of bioactives, contained in the reviewed aromatic plants. (**a**): Hydroxybenzoic acids, hydroxycinnamic acids and derivatives, phenolic diterpenes, triterpenic acids, phloroglucinols, and naphthodianthrones. (**b**): Monoterpenes, crocins, and flavonoids.

Flavonoids, phenolic acids, and phenolic diterpenes are the aromatic plant constituents mostly associated with their antioxidant properties. Other groups of compounds with bioactive properties are the phloroglucinols and the naphtodianthrones. In addition, apocarotenoid glycosides have exhibited valuable biological activities. The main structures of these compounds are presented in Figure 1a,b and it is evident that most of them contain phenolic hydroxyl groups. Besides their antioxidant capacity, many of these compounds present antimicrobial and antiviral properties and various beneficial health effects.

The Lamiaceae family contains several genera that have been extensively studied for their antioxidant potential. Outstanding among them is rosemary that is rich in carnosic acid, carnosol, and rosmarinic acid. Other Lamiaceae herbs, rich in rosmarinic acid and various flavonoids, are oregano species, lemon balm (*Melissa officinalis*), and *Satureja* species. All of them are easily cultivated and commercialized as spices. Additionally, their essential oil (EO) is used in cosmetics, food, and feed products. EO is obtained through steam- or hydrodistillation. Nevertheless, the yield of distillation is rather low, while the solid residue is discarded and causes environmental concerns, although it is a potent raw material for the recovery of antioxidants [2,3]. For example, rosemary yields around 0.3–2.5 g EO/100 g of dry plant [4,5,6,7], while a considerable amount of solid residue remains (10–20 × 10^3^ Tn/year) that is currently unexploited [8]. Moreover, deoiling leaves a raw material free of odors, and facilitates the phenolic compounds extraction probably due to enhanced penetration of solvent and mass transfer phenomena [4,9]. Therefore, this residue can be efficiently used for antioxidant extraction.

Another family is Hypericaceae, with most known among its genera the *Hypericum perforatum* (St. John’s wort). *H. perforatum* contains several flavonoids but also phloroglucinols (hyperforins) and naphtodianthrones (hypericins) that have been broadly examined for antioxidant activity and health effects the last decades. The plant has been traditionally used as a food supplement but also as a mild remedy. *Crocus sativus* (saffron) is another valuable herb, belonging to the Iridaceae family, which contains glycosylated C_20_ apocarotenoids, named crocins. Crocins are water-soluble compounds and have been recently associated with the treatment of various pathological disorders. The stigmas of the plant contain also safranal, the main aromatic compound that defines *C. sativa* as one of the most precious spices, and picrocrocin, a bitter taste glucoside of C_10_ apocarotenoid.

The present paper focuses on rosemary, oregano, pink savory, lemon balm, St. John’s wort, and saffron, as they are six common herbs with distinctive antioxidant bioactive components. The solvents and the methods used for the recovery and isolation of these components have been extensively reviewed. These methods often depend upon the particular class of antioxidant compounds and, to a lesser extent, on the nature of the matrix, but in general include solvent extraction, and may incorporate precautionary measures to protect the phenolic compounds, or green solvents to be environmentally friendly. Finding effective and feasible methods for the separation and purification of natural extracts rich in bioactive antioxidant compounds is a challenge for their industrial production and commercialization.

## 2. Rosemary

Rosemary (*Rosemarinus officinalis* L.) belongs to the Lamiaceae family and is native to the Mediterranean region and part of Asia, but can withstand cool climates and drought. Its name derives from the Latin ros-marinus, meaning “dew of the sea”, because it was believed to survive with no watering, only with the dew coming from the sea. It is the most well-known plant with antioxidant activity and its extract is the only currently approved natural antioxidant in EU (Directive 95/2/EC), assigned the E number E-392 (European Union Directives 2010/67/EU and 2010/69/EU). The antioxidant potency is primarily attributed to the phenolic diterpenes, carnosic acid and carnosol, and secondly to rosmarinic acid (and possibly other hydroxycinnamic acids, like caffeic acid), and minor flavonoid constituents (Figure 1). For this reason, the commercially available formulas of E-392 are standardized according to their content in carnosic acid and carnosol. The same constituents have been associated with several antifungal, antimicrobial, bioplaguicide, anticarcinogenic, anti-inflammatory, and prophylactic effects of rosemary extracts [8,10,11,12,13,14]. Rosemary and some common salvia species are the only herbs that contain carnosic acid and carnosol as major constituents [15]. Other compounds derived through carnosic acid and carnosol degradation like rosmanol, epirosmanol, epirosmanol ethyl ether, rosmadial, and methylcarnosate may be also present in the extracts [16,17]. The presence of triterpenoid acids, i.e., ursolic and oleanolic has been also reported [18]. The main flavonoids of the plant are apigenin, luteolin and other flavones, found mostly as glucosides [17,19,20,21]. All the identified compounds reported in the literature are presented in supplementary material (Table S1).

Carnosic acid and carnosol are compounds of medium polarity and therefore are effectively extracted with acetone or ethanol [21,22,23,24,25]. Other non-polar solvents like hexane and butanone proved also effective [24,25]. The extraction of carnosic acid in a shaking bath was enhanced with temperature (25–50 °C) and time (30–180 min), while butanone was more effective than ethanol, due to lower polarity [24]. The presence of water in mixtures with organic solvents decreases the extraction yield [21]. Confirming this observation, the fresh plant material presented lower carnosic acid extraction yield than the dried material due to the presence of water that combined with ethanol, which was used as solvent, resulting in a more polar solvent [24,26]. Additionally, carnosic acid is oxidized to carnosol and derivatives during extraction in the presence of water [27]. Comparing ethanol and methanol as solvents, it was found that the less polar ethanol is effective for the extraction of carnosic acid, while methanol for rosmarinic acid [26]. Water is an excellent solvent for rosmarinic acid, while increasing the organic solvents concentration in water decreases its extraction yield [21].

De AR Oliveira et al. [22] examined acetone, methanol, ethanol, and their mixtures with water for the quantitative recovery of rosmarinic acid, carnosol, and carnosic acid, and observed that ethanol 59–70% or acetone 80% gave similar results, while methanol 50% presented lower carnosic acid recovery due to its transformation to carnosol. Consequently, they used a central composite design to optimize the conditions for the simultaneous extraction of the three compounds with ethanol–water mixtures. Optimum conditions were defined as 70% ethanol in water, at a solid to liquid ration of 1:5, and extraction time 55 min, to recover 90% of the antioxidants, while achieving a high purity of the extract. Additionally, ethanol concentrations varying between 30% and 96% were tested in maceration experiments and 50% ethanol in water showed the highest phenolic yield and antioxidant activity [28]. Ethanol–water mixtures are considered green solvents and, therefore have been used by other researchers [4,29]. Psarrou et al. [21] examined ethanol or acetone mixtures with water and observed the highest total phenolic content (TPC) recovery, antiradical activity, and extraction selectivity with either ethanol 60% or acetone 60%. Mixtures of organic solvents with water are more effective than pure water because they can extract more quantitatively non-polar, e.g., phenolic diterpenes and flavonoid aglycones, plus polar compounds (phenolic acids and flavonoid glycosides). Furthermore, they examined the extraction kinetics and observed a fast initial extraction stage, followed by a much slower one, both of them following the unsteady state diffusion law. The increase of temperature (22–60 °C) enhanced swelling of the raw material, solubilization and diffusion of the solutes, thereby, and increased the extraction rate, but decreased selectivity as more non-flavonoid compounds were simultaneously extracted. Total terpenoids recovery increased with temperature but a high portion of carnosic acid was transformed to carnosol at 60 °C [21].

The main research results about the effect of extraction solvent and procedure are summarized in Table 1. Apart from the conventional solvent extraction (CSE), novel extraction methods, and, among them, ultrasound assisted extraction (UAE), has been examined by many researchers. UAE decreased extraction time and lead to more effective extraction, at lower temperature with less dependence on solvent [23,24]. In particular, it was found to markedly increase the efficiency of ethanol to extract carnosic acid and to enhance the antioxidant activity of the extract [24,26]. Both the extraction rate and the TPC yield increased by UAE compared to conventional solid liquid extraction performed under the same conditions, and the difference was more pronounced when ethanol 60% in water was used as a solvent instead of acetone 60% [21]. The fact can be explained by the lower penetration and solubilization ability of ethanol that is enhanced by UAE. Ultrasound intensifies mass transfer, due to collapse of cavitation bubbles near the cell walls that causes partial destruction of the cell walls and production of an ultrasonic jet, which may act as a micropump that can force solvent into the cell and dissolve the solutes [24]. Thus, UAE resulted in a meaningful shortening of processing time at about 10–12 min [21,30,31].

Bellumori et al. [25] examined UAE with different solvents in single or successive extraction steps. Ethanol and acetone gave the highest TPC yield, while water the lowest due to its inability to extract terpenoids, although it was the most effective for the recovery of rosmarinic acid and flavonoids. Additionally, sonication of water results in the formation of highly reactive hydroxyl radicals, which may participate to degradation reactions. The highest terpenoid recovery was obtained with acetone, accompanied with very limited oxidation of carnosic acid. Hexane presented low overall yield but a very high selectivity in terpenoids extraction. Thus, the authors concluded that UAE can be very favorably compared with the CSE in acetone that is used to prepare commercial rosemary antioxidants [25]. The investigation of the optimal conditions for the extraction of rosmarinic acid, ursolic acid, and oleanolic acid from rosemary leaves by UAE or maceration (90% ethanol, 48 h) indicated UAE with 70% ethanol the most efficient for rosmarinic acid recovery, UAE with 90% ethanol for ursolic acid, and maceration for oleanolic acid. Maceration showed also the highest TPC yield and antioxidant activity [18]. UAE performed with a probe presented higher extraction yield and carnosic acid and ursolic acid recovery, compared to a bath, possibly due to a better ultrasonic power delivery [29]. The results obtained at 40 °C for 30 min were comparable or slightly better than those obtained by conventional extraction at 78 °C for 30 min, except for rosmarinic acid that presented lower yield [29]. It is generally recommended to use reactors with 20 kHz as operating frequency in the case of UAE with a probe because, at lower frequencies of irradiation (e.g., 20 kHz), the physical effects of ultrasound-induced cavitation phenomena, i.e., liquid circulation currents and turbulence that are the controlling factors in extraction, are dominant [4,24,25,29,36].

Microwave assisted extraction (MAE) has been also examined [25,29,32,33]. MAE performed with water resulted in lower TPC yield than a conventional heat reflux extraction, while this was not observed when water mixtures with acetone, methanol, or ethyl acetate were used [32]. Water has a high dielectric constant but a low dissipation factor, compared to the other solvents. Thus, the rate of microwave energy absorbance is higher than the rate of heat dissipation, resulting in overheating and possibly destruction of some of the phenolic compounds [32]. Mixtures of methanol or acetone with water (70:30) presented the highest TPC yield [32,33], while mixture of ethanol with water (70:30) proved the most efficient for flavonoids [25,33], and, when acidified with 1% HCl, for anthocyanins [33]. The increase of temperature (78–150 °C) in MAE with 90% ethanol increased the extraction yield and rosmarinic acid recovery but decreased carnosic acid and ursolic acid recovery. Additionally, the use of vapor or N_2_ pressure was examined but did not enhance extraction yield [29].

Solid free microwave extraction (SFME) has been used mainly for the recovery of EO [9,37,38]. The principle of the method is the internal heating of the in-situ water of the plant by microwaves, which leads to rupture of the glands and oleiferous receptacles. The released EOs and bioactive compounds are evaporated with the in situ water of the plant material. If SFME is performed under pressure, at high temperature (around 180 °C), the polarity and viscosity of the water decrease and it can dissolve, and consequently, extract less polar compounds like flavonoid aglycons that are not soluble at atmospheric temperature and pressure [38].

Another approach used for the extraction of antioxidants is the accelerated solvent extraction (ASE) that is also defined as pressurized liquid extraction (PLE), and in case water is used as the solvent, pressurized water or pressurized hot water extraction (PWE, PHWE), or subcritical water extraction (SWE). Similar to UAE and MAE, ASE has several environmental and economic advantages compared to CSE. It is a fast extraction technique, requiring lower amounts of solvents, while non-toxic solvents like ethanol or water can be effectively used. In particular, when applying ASE with water, the polarity of water decreases as temperature increases while it remains at the liquid stage, thus it approaches the properties of organic solvents [34]. Ethanol proved a good solvent for the recovery of carnosic acid and carnosol by ASE, while rosmarinic acid was equally recovered by either ethanol, or water, and more polar acids (caffeic, chlorogenic) and flavonoid glycosides by water [19,34]. High temperatures, 150–200 °C, which may be used in ASE, cause degradation of rosmarinic acid [30,34]. Rosmarinic acid may be cleaved to its monomer, caffeic acid, which increased as temperature increased [30]. Additionally, increasing temperature caused an increase in gallic acid, while carnosic acid and carnosol were not affected, and consequently antioxidant capacity was favored. Nevertheless, as temperature increased, melanoidins were formed through Maillard reactions, which may lead to harmful products, thereby ASE at 150–200 °C was not recommended [30].

Additionally, supercritical fluid extraction (SFE) has been examined by some researchers. SFE with neat CO_2_ provides very low yield that can be improved with the addition of a modifier such as ethanol [19,39,40]. In fact, CO_2_, as a non-polar solvent, can recover only carnosic acid, carnosol, and other carnosic acid derivatives, even at 400 atm, while the addition of 7% or 10% ethanol was necessary for the extraction of minor amounts of other phenolic compounds [19,34,40]. Zabot et al. [41] proposed a sequential extraction of the EO and the phenolic compounds by using supercritical CO_2_ and PWE in the same equipment. Water is a polar solvent, thus suitable for rosmarinic acid extraction that was recovered at the beginning of PWE. As temperature increased above 100 °C, the polarity of water was reduced and the less polar compounds, i.e., carnosic acid, carnosol, rosmanol, and methyl carnosate were obtained [39,41].

Another research team proposed a pressurized hot water extraction combined with particle formation on line (WEPO) to obtain dry antioxidant powder from rosemary [34,42]. The extraction is performed at 200 °C and 80 atm, the extract is continuously transformed to an aerosol by the use of a supercritical CO_2_ nebulization system, and the aerosol is instantaneously dried by a hot N_2_ current [42]. After 40 min of extraction a powder yield of 34%, dry basis, was obtained with good DPPH radical scavenging properties, while no details about the phenolic profile are provided by the authors. The procedure was favorable in terms of environmental impact, compared to PHWE (200 °C, 103 atm, 20 min) and SFE (40 °C, 150 atm, 300 min, ethanol as modifier) giving powder with similar antioxidant capacity. Additionally, ionic liquids have been examined, as novel, green solvents [43] but separation of the antioxidant compounds from the extraction liquor needs further research.

The plant material is dried (usually at room temperature) before the extraction so as to avoid microbial spoilage during storage and facilitate transportation. Mulinacci et al. [27] observed that drying caused a significant loss of flavonoids and rosmarinic acid, while total terpenoids were not affected. Additionally, freeze drying caused significant losses [27,44]. Freezing, on the other hand, caused a high loss of rosmarinic acid, possibly due to phenoloxidase activity [27]. On the contrary, grinding of the raw material to smaller particle size, facilitated mass transfer phenomena, and consequently, enhanced extraction [21]. The geographical region, and possibly the soil type, and altitude have an effect on the profile and concentration of the phenolic compounds [20,45,46]. The harvesting period has a significant effect on the phenolic content that presents a maximum on flowering period (e.g., May and November), and on flavonoid content that follows the same trend [44]. Furthermore, the highest concentration of carnosic acid and rosmarinic acid present a reverse trend, the former showing a maximum in summer and the latter in winter [45]. However, results for phenolic compounds seasonal variations from plants of different regions and countries do not agree and seem to depend, among others, to variations in temperature and rainfall [43,46,47].

Rosemary extracts have been proposed and used as bioactive, antioxidant additives in food, cosmetics, packaging, etc. [48,49,50,51,52]. Their worldwide market is expected to present an annual growth rate of roughly 3.7% over the next five years, and will reach 260 million US$ in 2024 from 210 million US$ in 2019 [53]. For industrial uses dried extracts have several advantages, e.g., they are easier to handle, transport and store, and to be used in solid formulations like tablets and capsules. Dried extracts have been obtained through spray drying of an ethanol:water (80:20) extract at an inlet temperature of 140 °C. Although the dried products lost some of their polyphenols, they presented appreciable antioxidant activity [54]. Other investigators reported much lower inlet temperature (80 °C) as optimum [55]. Efforts for encapsulation in maltodextrin, through spay drying, presented promising results, too [35].

## 3. Oregano

Oregano is one of the most common and important aromatic and medicinal herbs of the Lamiaceae family. Thousands of tons are consumed every year as a spice and its flavor is highly favorable all over the world. The food industry uses dry oregano as a spice in snacks, salad dressings, etc., where in addition to the desirable flavor it may provide antioxidant protection. Many studies have pointed out the antiviral, anti-inflammatory, and antitumor properties of its EO due to its high content in carvacrol and thymol [56,57,58]. Although rosemary extract is the only one, among Lamiaceae herb extracts, approved by EU legislation as food antioxidant (additive), the US Food and Drug Administration (FDA) has also recognized oregano EO as a safe and potable substance (generally recognized as safe—GRAS) [59]. Additionally, carvacrol has been approved for food use by the European Union (Commission Implementing Regulation (EU) No 872/2012).

Many oregano plants are widely used under the vernacular name oregano. Although similar in their external appearance, differ in their odor and consequently in their composition. At least 60 species and 17 genera belonging to diverse botanical families are known as oregano [60]. The most common is *Origanum vulgare* L. (Greek oregano). Oregano population are primarily distributed in Eurasia and African regions, with the highest recorded diversity being in the Mediterranean [61]. Various methods have been used to differentiate extracts of *Origanum* subspecies from different part of the world, or to isolate and characterize new phenolic compounds [62,63].

Several studies [64,65,66,67,68] have reported oregano as one of the most promising sources for the recovery of polyphenols and consequently for its antioxidant properties, which have been proved to be highly dependent on the total phenolic content. Considerable amounts of phenolic compounds are generally detected in extracts obtained with water, methanol, ethanol, acetone, ethyl acetate, and/or mixtures of them, by using conventional or novel methods. Flavonoids (mainly apigenin, luteolin, quercetin, and their glycosides), and phenolic acids (mainly rosmarinic acid) are the main types of bioactive compounds present in oregano (Table S1) [69,70,71,72,73]. Rosmarinic acid appears the major phenolic component of oregano and its maximum content can be near 23 mg/g of plant material [74]. A study among oregano herbs of different origin, such as Turkish, Syrian, and Spanish, showed that Syrian is the richest one in rosmarinic and caffeic acid [75]. Additionally, lithospermic acid (and its stereoisomers) was isolated from *O. vulgare* spp. *hirtum*, [64,73]. The high antioxidant capacity (DPPH, ABTS, and FRAP) of the *O. vulgare* methanolic extract was attributed to the large quantity of rosmarinic acid (23.53 mg/g of dry extract) and the presence of other active compounds like (−)-epicatechin [76].

The first attempts of obtaining oregano extracts with high antioxidant activity were based either on Soxhlet extraction [67,77], or CSE [68], by using solvents of different polarity. *O. vulgare* water extract, among the extracts obtained by maceration with water, ethanol, acetone, ethyl acetate, or diethyl ether, proved to have the highest total phenolic content (235 mg GAE/g extract), while diethyl ether extract the highest flavonoid and tannin content (132 mg rutin equivalents/g extract, and 4 mg catechin equivalents/g extract, respectively). Additionally, water obtained the maximum TPC recovery (51 mg GAE/g dry plant) but ethanol, exhibiting a much lower recovery (9 mg GAE/g dry plant), had a higher antioxidant activity, indicating that ethanol was a more selective solvent for phenolic compounds and the obtained extract contained less impurities [70]. The successive extraction of *O. vulgare* with ethyl acetate, water, and ethanol led to a combined extract with high antioxidant activity, with quercetin glucoside and apigenin glucoside being the major components (49.72% and 23.70% of the total flavonoids, respectively) [66]. Mixtures of organic solvents with water have been studied for a more efficient quantitative and qualitative recovery of the desired compounds, than pure solvents, as they can extract rosmarinic, caffeic and other phenolic acids, and the less polar flavonoids and even carvacrol. The use of 80% methanol in water extract indicated a lower content of phenolic compounds compared to the water infusion and decoction, while the analysis revealed the presence of 22 phenolic compounds, with rosmarinic acid being the most abundant phenolic acid (15 mg/g extract) in all the preparations, and luteolin 7-*O*-glucoside and luteolin *O*-glucuronide being the most abundant flavonoids (12–28 mg/g extract) [69]. Majeed et al. [78] examined the effect of methanol in aqueous mixture (70–90%) and found that 70% methanol resulted in the highest TPC recovery (18.8 mg GAE/g dry plant after 16 h of extraction) from *O. vulgare* leaves, accompanied with the highest DPPH radical scavenging capacity. *O. vulgare* spp. *hirtum* 70% aqueous-methanol extract, obtained by UAE, contained a high amount of total phenolics (49.9 mg GAE/g extract, dry basis), rosmarinic acid (12.0 mg/g extract), and carvacrol (28.8 mg/g extract), and a significant amount of (±)-naringenin and rutin [79]. As ethanol presents similar yields to methanol, it can be used instead of the latter for food or cosmetic uses of the recovered polyphenols, as it is less toxic. *O. vulgare* spp. *hirtum* extracts obtained with 60% ethanol exhibited the highest phenolics recovery (both phenolic acids and flavonoids) and the strongest antiradical activity [64]. In particular, the increase of ethanol content from 0% to 60% showed increase in the yield of rosmarinic and lithospermic acid.

The antioxidant capacity of *O. vulgare* is not entirely related to rosmarinic acid, which indicates that other compounds are acting as antioxidant agents [80]. A positive correlation was observed between the TPC and DPPH radical scavenging activity and was attributed to the presence of eriodictyol, apigenin and caffeic acid in the aqueous extract of *O. vulgare* [81]. Furthermore, eriodictyol and naringenin were also found in the methanolic extract of *O. vulgare* leaves, which exhibited high TPC and a positive correlation with the ORAC value [82].

Changes in the extraction process variables (solid-to-liquid ratio, temperature) can affect both the TPC yield and the phenolic profile of an extract. A solid/liquid of 1/20 proved efficient for the recovery of phenolic antioxidant components from oregano [64,78]. Lower values (e.g., 1/40) did not increase the extraction rate or TPC recovery [64], while values higher than 1/12.5 decreased sharply the TPC recovery in CSE [78]. Experiments with oregano extracted by ethanol at various temperatures revealed that TPC increased as temperature increased in the range of 20–60 °C [64].

Phenolic compounds are found in both free and bound forms in plant cells. The free phenolics are easily extracted. On the contrary, phenolic compounds covalently-bound to the plant matrix cannot be extracted by water or organic solvents, thus alkaline or acid hydrolysis is needed [2,83]. *O. hirtum* extraction using KOH 1% or 3% showed noticeable antiradical activity, with rosmarinic acid content amounting to 5.02% and 4.66% in the dry extract, respectively, indicating a possible degradation of rosmarinic acid to caffeic acid, as the latter increased in the extract obtained with 3% KOH [2].

Several investigators explored the use of novel methods (ASE, UAE, and SFE) for the extraction of bioactive compounds from oregano, in an attempt to reduce extraction time and solvent and thereby approach green extraction techniques. The main results are summarized in Table 2. By using aqueous methanol mixtures at 103 atm in ASE, oregano showed its optimum extraction condition at 33% methanol and 129 °C, with the ASE extracts having significantly higher amount of rosmarinic acid (10.21 mg/g) than the CSE extracts (5.70 mg/g) [30]. It is interesting to note that the optimum methanol concentration was lower than that defined for rosemary extraction (56%), while the temperature was the same because at higher temperature degradation of luteolin and apigenin glycosides was observed. Rodríguez-Meizoso et al. [84] performed ASE with water at 103 atm and temperature varying from 25 to 200 °C. The yield and phenolics recovery increased with temperature, while the antiradical activity of the extracts obtained at elevated temperature was higher due to the presence of different phenolic compounds (mainly flavonoids).

Comparing SFE and focused UAE (ultrasound energy is focused in the tip of the ultrasound probe) techniques, the TPC obtained by means of focused UAE was higher and so was the antioxidant capacity of the extract [85]. However, the study of the recovery of carvacrol, rosmarinic, oleanolic, and ursolic acid from three different oregano species (*O. onites* L., *O. vulgare* spp. *hirtum,* and *O. vulgare* L.), with alcoholic mixtures, by using various extraction techniques, showed that heat extraction (95 °C) under reflux, and continuous stirring extraction at ambient temperature, gave significantly higher values, compared to percolation, maceration and UAE, while heat extraction needed shorter time [65]. The extracted maximum rosmarinic acid amount from *O. vulgare* ssp. *hirtum* was 3.85 times higher than *O. vulgare* L. and 2.2 from *O. onites* L. Similarly, water extracts obtained from *O. vulgare* at 85 °C revealed higher TPC and antioxidant activity than the ones obtained by UAE at room temperature [87].

SFE was applied in order to obtain extracts from *O. heracleoticum* rich in compounds with antioxidant activity but free of the lower molecular weight aromatic compounds. Fractionation by applying 100 atm at 40 °C, followed by 300 atm at 40 °C, and 300 atm at 100 °C, resulted in a partial separation of the components, with a higher content of EO components in the first fraction, and thymoquinone and a low flavonoid content in the third, which exhibited the strongest antioxidant activity [88]. Furthermore, the addition of ethanol, as a cosolvent, improved the efficiency of SFE and enhanced the coextraction of polar compounds [85,86]. Medium polarity molecules, such as the flavonoid aglycons dihydroquercetin, eriodictyol, and dihydrokaempferol, were only extracted with ethanol as modifier. The oregano matrix is relatively soft compared to rosemary, thus SFE with ethanol was more efficient for the extraction and fractionation of oregano flavonoids [86].

As the fresh herb is prone to microbial spoilage, drying facilitates its storage and also handling. Drying of oregano resulted in considerable increase in the recovery of TPC in comparison to extracts from fresh plant material, however the antioxidant activity against linoleic acid oxidation was not affected and the DPPH radical scavenging ability was reduced [89]. A study among different drying methods revealed that air-dried extracts of oregano had significantly higher rosmarinic acid content than the vacuum oven-dried, the freeze-dried and the fresh samples [90]. Additionally, grinding of the herb increases the contact surface area and shortens the diffusion path, and, thereby, increases the extraction rate. Thus, a particle size of <315 μm strongly affected the initial rate of extraction, and, consequently, decreased to more than half the time needed for the total phenolic extraction. However, the final recovery was not affected by the particle size [64]. Majeed et al. [78] observed that particle size had a smaller effect than methanol-in-water concentration, solid-to-liquid ratio, and extraction time, but still a higher TPC recovery was obtained as particle size decreased from 110 to 20 μm.

The standardization of extracts obtained from oregano is investigated in order to facilitate their integration into food, beverages, food preservatives, cosmetics, dietary supplements, pharmaceuticals, and nutraceuticals. Formulations such as liquids, powders, pastes, and gels are currently in the market [91]. Powders are mostly used as ingredients in dietary supplements such as tablets and capsules. These formulations are also intended to protect the sensitive oregano extract compounds against oxidation. Additionally, the encapsulation of the extracts in water-soluble microcapsules can protect physically the active components during storage and, moreover, during the digestion process, favoring the maintenance of their antioxidant activity [92]. Oregano extracts were effectively microencapsulated in maltodextrin and the concentration of phenolics and flavonoids was higher when 10% instead of 15% maltodextrin was used. The powders formed by encapsulation of the extract obtained by hot extraction were 1.6 times richer in total phenolics than those obtained by cold extraction [35]. Another potent use of oregano extracts is in active packaging, where it can be incorporated or coated in edible or non-edible packaging material. Together with tea and rosemary extracts, oregano extract is one of the most extensively examined for this application, with very positive results [52,93].

## 4. Pink Savory

Pink savory (*Satureja thymbra*) is a member of the genus *Satureja*, which consists of about 200 species, widely distributed in the Mediterranean area, Asia, and North America, regularly found in sunny, dry, rocky habitats [94]. *S. thymbra* extracts possess several components with antioxidant and pharmacological activities [95,96,97]. Additionally, the plant showed antiviral potential against SARS-CoV and HSV-1 infection [96,98]. *S. thymbra* EO is especially rich in oxygenated monoterpenes. Among these, the best known, are thymol and carvacrol.

The extracts of *S. thymbra* are rich in phenolic acids and flavonoid compounds (Table S1). Rosmarinic acid is the main phenolic acid, followed by salvianolic acid A and lithospermic acid, well-known caffeic acid derivatives [99]. Luteolin, apigenin, eriodictyol, and naringenin, together with ethers of luteolin and apigenin are the main flavonoids identified, while aromadendrin, taxifolin and ladanein have been also reported [79,100,101]. Apigenin-7-O-glucoside is a common glycoside in Lamiaceae, which has been identified by numerous researchers [73,102,103]. Tsimogiannis et al. [101] identified luteolin-7-O-rutinoside in the ethanol extract of *S. thymbra*. This compound has been also identified in *S. hortensis* and *S. montana* [102,104].

Research efforts for the extraction of antioxidant compounds from *S. thymbra* are limited (Table 3). Sequential Soxhlet extraction with ethyl acetate, and ethanol, of the by-product derived through water–steam distillation to recover the EO, indicated a TPC recovery of 154 and 289 mg GAE/g dry plant, respectively, while the water remaining in the distillatory had an additional 249 mg GAE/g dry plant. All the extracts showed high DPPH radical scavenging capacity, following the order ethanol extract > water > ethyl acetate extract [101]. Among the byproducts of the EO hydrodistillation from several Lamiaceae family plants, namely *S. thymbra*, *O. dictamnus*, *O. hirtum*, *O. onites,* and *R. officinalis*, subjected to mild alkaline extraction, *S. thymbra* extracts showed the best antiradical activity, and the highest content in rosmarinic and caffeic acid [2]. These results show that the waste from *S. thymbra* EO production can be exploited for the recovery of antioxidants.

Özkan and Özcan [107] used mild acidic hydrolysis to release bound phenolic compounds during the extraction with mixtures of organic solvents with water. Thus, they compared four different solvent mixtures (ethanol:water:acetic acid (95:4.5:0.5), methanol:water:acetic acid (95:4.5:0.5), acetone:water:acetic acid (95:4.5:0.5), and methanol:acetone:water:acetic acid (55:40:4.5:0.5)) using either Soxhlet extraction or UAE (bath). The highest TPC and antioxidant properties were determined in the extract obtained using ethanol:water:acetic acid (95:4.5:0.5) in Soxhlet. The extracts showed strong antioxidant activity as measured by the phosphomolybdenum method in vitro, by their capacity to scavenge DPPH radical, and by their ability to decrease the rate of peroxide formation in olive oil in comparison to synthetic antioxidants, like BHA and BHT. Additionally, extraction by 70% methanol in water proved much more effective than water for the recovery of phenolic acids and flavonoids. The extraction was performed in an ultrasound bath for 20 min, at 30 °C and the predominant phenolic acid was rosmarinic, representing 88% of the total phenolic acids, while naringenin was the predominant flavonoid, representing 91% of the total flavonoids [79]. Rosmarinic acid was found in higher concentrations in *Satureja* species and *R. officinalis* compared to other Lamiaceae family plants [105].

Recently, three glycerol-based eutectic solvents were tested for their efficiency to recover polyphenolic antioxidants from *S. thymbra* [106]. The process was optimized by Box–Behnken design and response surface methodology (RSM) with respect to water concentration (optimum values 54.8–63.8%, *v/v*) and solid/liquid (optimum values 1/30–1/36 g/mL). Yields approximated 140 mg GAE/g dry weight, and the chromatographic analysis showed the presence of several phenolic substances, tentatively ascribed to rosmarinic acid, apigenin, luteolin, and quercetin derivatives. Although none of the eutectic mixtures showed selectivity, the mixture composed of glycerol and trisodium citrate was proposed for further research by the authors.

Ethyl acetate and ethanol extracts of *S. thymbra* proved effective antioxidants against olive oil or vegetable oil oxidation and also retarded significantly the oxidation of emulsions [101,107,108]. Additionally, the ethanol extract combined with the plant EO exhibited antioxidant and antimicrobial activity when added into a carboxyl-methyl-cellulose edible film that was used for fresh gilthead seabream fillets, by reducing the peroxide values by approximately threefold and eliminating the formation of secondary oxidation products [99]. Additionally, *S. thymbra* extracts proved effective when coated on non-edible film used as active packaging for snacks. More specifically, incorporated in the packaging they protected the snacks better than adding them to the frying oil or to the fried product [109]. These results show that *S. thymbra* is one of the most potent sources of antioxidant and bioactive compounds, while purification and formulation of its extracts need further research.

## 5. Lemon Balm

Lemon balm (*Melissa officinalis* L.) is a common plant of the Lamiaceae family, native to Europe, Central Asia, and Iran, but now growing around the word. It is used as flavoring in confectionery, teas, and certain foods, and in traditional medicine. Its EO is used as a perfume ingredient and in aromatherapy [110]. Additionally, the plant is used to attract bees, as it flourishes in summer, with white small flowers full of nectar, hence the name melissa, meaning bee in Greek. Several studies have demonstrated the antioxidant plus various biological activities, such as antimicrobial, anti-inflammatory, antiplatelet, anticancer, antidepressant, anxiolytic, hypolipidemic, etc. [79,110,111,112,113,114,115,116]. The main constituents associated with these activities are triterpenoid acids, phenolic acids, and flavonoids.

The main identified triterpenoids are ursolic and oleanolic acid and their derivatives [114,117]. Rosmarinic acid was the main phenolic acid in *M. officinalis* extracts, amounting to 1.50–6.8% of the dry leaves of the plant [111,113,118,119,120], and it was associated to the antioxidant properties of these extracts [114,117,121]. Additionally, rosmarinic acid showed antimicrobial activity, contrary to the triterpene derivatives that showed very low or no antimicrobial activity [114]. Compared to other Lamiaceae herbs, *M. officinalis* presents a very high content of rosmarinic acid [122,123].

Table 4 presents the main reported results about the solvents and extraction methods that have been used for the recovery of the plant bioactive components. Awad et al. [113] performed successive extractions with hexane, ethyl acetate, methanol, and water (solid/liquid, 1/10, *w/v*). Hexane extract showed no effect, while methanol extract exhibited the highest activity towards the inhibition of the rat brain γ-aminobutyric acid transmitase (GABA-T), an enzyme targeted for the therapy of neurological disorders, like anxiety and epilepsy. The extract was rich in rosmarinic acid, while ursolic, oleanolic, caffeic, and other not identified hydrocinnamic acids were present, and might exhibit additive or synergistic actions with rosmarinic acid. Rosmarinic acid was the most abundant phenolic compound in water infusions, alcohol, or alcohol–water extracts, according to several researchers, while salvianolic acids, lithospermic acid, caffeic acid, and their derivatives were also reported [79,105,119,123,124,125,126,127]. Gentisic, gallic, and small amounts of *p*-coumaric, protocatechuic, and chicoric acids were also detected [105,116,119,126,127,128]. Luteolin, luteolin-7-*O*-*β*-glucoside, and luteolin-3′-*O*-glucuronide were among the main reported flavonoids of the herb [114,117,124,125,126]. Quercetin, myricetin, epigallocatechin, and rutin were also detected in ethanol or water extracts [66,79,116,129]. The identified compounds reported in literature are presented in Table S1.

Since the phenolic compounds of *M. officinalis* comprise mostly phenolic acids and flavonoid glycosides, acetone, and especially hexane extracted very low amounts, contrary to ethanol that presented high TPC recovery and antioxidant activity of the extracts [140]. Aqueous alcohol mixtures were further examined by several researchers. Aqueous ethanol and water were much more effective than pure ethanol in the extraction of phenolic compounds [141]. Methanol–water, ethanol–water, and water were compared for their efficiency in the quantitative recovery of rosmarinic acid. Both alcohol mixtures were more efficient than water and the recovery increased when the mixtures were acidified [119,133]. Caffeic acid and protocatechuic acid were also determined in the extracts. Three successive extraction steps with methanol 60% in water solution, at a solid/liquid of 1/100, g/mL, for 10 min each, by the use of UAE at 25 °C, were sufficient for the quantitative recovery of all acids [119]. Successive extractions are necessary because a large amount of the extract (about 40% of the initial solvent mass) is retained in the herb [120]. Sik et al. [133], using lemon balm and other herbs, reported either 70% or 50%, both of them acidified with 1% HCl, as optimum methanol concentration in aqueous solutions, depending on the herb and extraction method. Optimization of rosmarinic acid extraction with methanol in water mixtures, by RSM, revealed 59% methanol as the optimum concentration, while increase above 65% resulted in lower yield [132]. Similarly, testing different ethanol in water concentrations the highest rosmarinic acid yield was obtained with 50% [120,133,134], or 70% if acidified with 1% HCl [133], while 30–60% gave the highest extraction yield for both rosmarinic and caffeic acids [131]. Rosmarinic, caffeic and other phenolic acids are soluble in both water and aqueous alcohol solutions. Thus, the higher effectiveness of the latter in quantitative phenolic acids extraction might be attributed to a better penetration in the plant matrix. Rosmarinic acid and other extracted antioxidants can be separated from the ethanol–water mixtures by nanofiltration, instead of evaporation, and the permeate can be recycled, with apparent economic benefits [120]. The procedure has been successfully tested in both lemon balm and rosemary extracts and the retentate maintains its antioxidant capacity and can be used directly as preservative or functional ingredient in foods, cosmetics, or medicines, as it presents a high concentration of active compounds [120,142].

Optimization of the water extraction conditions resulted in 100 °C for 120 min as the optimum conditions for TPC recovery and antiradical (DPPH, ABTS^+^) activity of the extracts [138]. Hydrolysis with 0.2 M hydrochloric acid increased the TPC content of the extract, and the FRAP, indicating that glycosylated forms might have been hydrolyzed to the respective aglycons that present higher antioxidant power. The content in rosmarinic acid was not significantly affected by hydrolysis, on the contrary the content in caffeic acid was increased by approximately 10-fold [143].

During CSE with ethanol–water mixtures, the yield increased rapidly at the beginning, slowing down towards the end of the extraction [120,130,134]. The kinetic study, based on the extraction yield, indicated that after an initial spontaneous extraction, a fast and a slow extraction stage followed, obeying to non-steady diffusion as described by the 2nd Fick law [130], similarly to the observations for rosemary and oregano [21,64]. Grinding of the raw material to smaller particle size, and increase of solvent in the mixture had a minor effect on the fast extraction stage but increased the rate of the slow stage and the final yield. Extraction temperature (0–80 °C) had a variable effect on the detected phenolic acids (carnosic, ursolic, and oleanolic), possibly due to the very low detectable concentrations and the sensitivity of these compounds to higher temperatures [130]. Another study demonstrated that the increase of temperature from 25 to 50 °C caused a minor increase in rosmarinic acid yield obtained by aqueous ethanol, while further increase to 60 °C had no effect [134]. On the contrary, a significant increase from 25 to 55 °C was observed when aqueous methanol was used as solvent [132]. A pretreatment by SFE removed EOs, waxes, and chlorophylls and thus changed the structure of the plant material, facilitated the access of extraction solvent, and, thereby, increased the extraction rate and the rosmarinic acid yield [120,144]. The increase was higher when the SFE was performed at harsher conditions (higher pressure and temperature [120]. These results indicate that the conventional extraction of antioxidants can be favorably combined with the EO extraction by SFE. However, longer SFE duration or higher pressure may lead to the extraction of triterpenes and flavonoids and consequently decrease the antioxidant compounds in the residue [144], while the ethanol addition as modifier decreased also rosmarinic acid content in the residue [120].

UAE enhances the extraction rate and thus may decrease the extraction time to as low as 10 min [119,131] compared to approximately 100 min for CSE under agitation [120,130,134]. Considered a green extraction technique, it has been used in extraction performed in short time for analytical purposes [145]. Although UAE at room temperature with either water or ethanol 25% presented lower phenolic content in the extract and FRAP values, than the obtained in infusion preparations (10 min) and mostly decoctions (boiling for 10 min), the DPPH radical scavenging capacity did not show significant differences, indicating that rosmarinic acid and other phenolic compounds reacting with DPPH were equally extracted by either method [141]. In fact, UAE performed with ethanol showed a low extraction yield but a high selectivity in the extraction of phenolic compounds and rosmarinic acid [136].

Caleja et al. [135] used experimental design and RSM to study the effect of ethanol concentration, extraction time, and temperature or power in CSE under heating, UAE, and MAE. They found that all variables were significant for the rosmarinic acid recovery. The most efficient method was UAE performed at 30–35 °C, with optimum yield 86 mg/g dry plant, at 40% ethanol, 371 W, and 33 min. Lower ethanol concentrations were defined as optimum for the CSE under heating and MAE (34.5% and 25.5%, respectively), with optimum temperatures 88 and 108 °C, respectively, and extraction time 106 and 26 min, respectively. According to these results MAE was the fastest extraction method but gave the lowest rosmarinic acid recovery (49 mg/g dry plant), and CSE the slowest method giving optimum recovery equal to 59 mg/g dry plant. Changing solid/liquid from 1/10 to 1/200, g/mL, increased the extraction yield, up to 153 mg/g dry plant for the optimum UAE conditions [135]. When water was used as solvent MAE obtained higher TPC and rosmarinic acid yield, compared to UAE and conventional extraction [139]. These differences may be attributed to the solvent and also the different equipment and power used by the researchers. Similarly, MAE performed at mild temperature (100 °C) under inert atmosphere (N_2_) with either ethanol or water gave better results than UAE [136], indicating that the extraction conditions play a major role in the protection of the phenolic compounds from degradation. The highest extraction yield was obtained with water, but the highest total phenolic and rosmarinic acid yields with ethanol, which proved a much more selective solvent for the phenolic compounds. Additionally, UAE successive extractions with solvents of increasing polarity were tested, and the results indicated that hexane had no effect, while acetone improved the efficiency and selectivity of the following extraction with ethanol [136], probably due to removal of waxes, EO constituents, etc., and thus making the plant matrix more accessible.

Sik et al. [133] compared MAE (at 50 and 80 °C) with maceration under stirring and CSE at boiling temperature, with aqueous ethanol and methanol for the extraction of rosmarinic acid from lemon balm, rosemary, oregano, and other herbs. They concluded that MAE was only superior to conventional methods with respect to extraction time (5 min for MAE, compared to 15 min for CSE under heating, and 120 min for maceration). Moreover, they tested various ethanol and methanol concentrations in water and observed some differences depending on the herb and the extraction method, but generally 70% ethanol gave very good results in all cases. Radomir et al. [137] used in vitro cultured plants and examined MAE with ethanol, 70% and 96% for the recovery of total phenolic compounds. Ethanol 70% was more effective, while the recovery increased up to 10 min of extraction and decreased afterwards, possibly due to compounds degradation. Higher temperature, i.e., 60 °C, increased the extraction efficiency, compared to 25 and 40 °C that presented the same results.

ASE (150 °C, 20 min static extraction time) tripled the TPC of the extract compared to CSE (50 °C, 2 h, pH 5). Water was more effective than ethanol as a solvent in ASE (TPC: 193 mg GAE/g extract, versus 167 mg GAE/g extract) [127]. Enzymatic assisted extraction (EAE) with combinations of cellulose, *β*-xylanase, and pectinase was also tested, and slightly increased the TPC compared to CSE (TPC: 79 mg GAE/g extract versus 65 mg GAE/g extract) [127]. The antioxidant activity followed closely the TPC. These results indicate that the bonding of phenolic compounds is more sensitive to temperature than to acidity or enzymatic treatment. On the contrary, the extraction yield (g dry extract/g dry material) obtained by EAE was higher than ASE with water and non-enzymatic treatment, while ASE with ethanol presented more than 5-fold lower yield, indicating that a high amount of non-phenolic compounds was extracted by the former treatments. Consequently, ASE with ethanol provided the most selective extraction of antioxidants.

Milevskaya et al. [126] obtained the highest phenolic compounds recovery from *M. officinallis* and other Lamiaceae herbs, by ASE, compared to CSE under heating, UAE, or MAE.

Drying of the herb (room temperature 10 days) and storage for 6 months did not affect the TPC or the DPPH scavenging activity of the obtained extract [89]. Cultivated and especially in vitro cultured samples contained less than half TPC than the dried commercial samples, possibly due to the lower production of secondary metabolites by the plant, when grown without stress [124]. Examination of different cultivars and seeds from different companies revealed that they play an important role in TPC, phenolic profile, and antioxidant properties of the herb. Especially rosmarinic and gentisic acids were significantly affected by the cultivar [128]. Moreover, the harvesting period and time have a significant effect on the accumulation of bioactive compounds, with starting of blooming period (early June) and afternoon showing the maximum amount [146].

*M. officinallis* extracts have demonstrated antiradical/antioxidant properties in various systems, including *β*-carotene-linoleic acid bleaching, superoxide anion, nitric oxide and DPPH radicals scavenging, and ferric chelation, indicating that they have the potential to prevent oxidative damage in vivo and pathological disorders [145,147]. Additionally, they proved effective antioxidants for lipid protection in oils and emulsions [112]. Water extracts, rich in phenolic compounds were effectively encapsulated in maltodextrin, by spray drying [138]. However, there is a need for further study towards the purification and formulation of these extracts in order to be exploited commercially.

## 6. St. John’s Wort

St John’s wort (*Hypericum perforatum*) belongs to the Hypericaceae family and is abundant in Europe, part of Asia, North America, and Australia. In addition to antioxidant compounds, St. John’s wort extracts possess several components with pharmacological activities [148,149,150]. The plant extracts are traditionally used against mild depression and for the treatment of infected wounds. Most of the pharmacological activities, and especially the antidepressive activity, were initially attributed to hypericins but recent studies revealed that hyperforins and the flavonoid components contribute to this activity, possibly through synergistic actions [148,151,152]. However, although the plant is included in several Pharmacopeia, both FDA and EU council consider it a dietary supplement, and not a drug [152,153]. Overall, the bioactive constituents of the plant may be classified in three main categories, phloroglucinols (mainly hyperforin and adhyperforin, Figure 1a), naphtodianthrones (primarily represented by hypericin and pseudohypericin, Figure 1a), and flavonoids (quercetin, quercetin glucosides, kaempferol, etc., Figure 1b). It is the only species that contains hyperforin as one of its main ingredients [154]. Additionally, the plant contains phenolic acids, such as chlorogenic, and carotenoids, while it is rather poor in EO (0.05–0.9%) [148,155]. The compounds, identified in higher quantities in the plant, together with their yields by several extraction procedures, are presented in Table 5. Additionally, the buds and flowers of the plant contain some protopigments, i.e., protohypericin and protopseudohypericin, which are transformed to hypericin and pseudohypericin under the exposure to light [156].

Hyperforin is the main phloroglucinol component of the plant and is considered as a potent antidepressant, remedy against inflammatory skin diseases of wounds, and antimicrobial agent [148,162,163,164]. It is a lipophilic compound and can be recovered by non-polar solvents, like diethyl ether, petroleum ether, and hexane [157,164,165]. Nevertheless, n-hexane presented very low recovery during long lasting Soxhlet extraction because hyperforin is strongly degradable in aprotic solvents [166]. Thus, it should be stabilized during and after the extraction by the addition of ascorbic palmitate or a mixture of ascorbic and citric acids, via transformation to weak salt by dicyclohexylamine base, or through transfer to methanol [164]. Moreover, it is unstable under heat, air, and light, indicating ambient temperature, absence of light and air as the best extraction and storage conditions, and as necessary precautions of analysis [159,165,167]. Furthermore, a 20% loss of the compound was observed when exposing the plant flowers to light for 2 h, while drying under dark had no effect [167].

Additionally, to non-polar solvents, methanol and ethanol have been proposed for the quantitative extraction of phloroglucinols (hyperforin and adhyperforin), though the extracts are also rich in hypericins [167]. Hypericins cause photosensitivity and are not desirable components in some cosmetic or pharmaceutical applications of hyperforin. For example, hypericin-free but hyperforin-rich products are proposed against inflammatory skin diseases, such as atopic dermatitis [168]. Therefore, the respective commercially available extracts for cosmetics are usually standardized according to their hyperforins content and to the absence of hypericins.

A hypericin-free extract of hyperforins can be obtained by supercritical or subcritical CO_2_ (70 atm, 22 °C). The latter proved the most selective solvent for hyperforins that amounted to 60% of the total extract weight [169]. Supercritical CO_2_ at 450 atm and 40 °C resulted in a recovery of 24.0 mg/g dry plant material [166], while at 100 atm and 40 °C a yield amounting to 19.0 and 2.9 mg/g dry plant material for hyperforin and adhyperforin, respectively, was reported [170]. In general, higher CO_2_ density, which was obtained by higher pressure and lower temperature, resulted in higher hyperforin yield [170,171]. Trying to optimize SFE conditions, Cui and Ang [163] reported that 380 atm at 50 °C presented the best results, and more than 95% of hyperforin and adhyperforin were extracted after 10 min static, followed by 1.5 h dynamic extraction, while the use of a co-solvent did not improve the results, on the contrary increased polar impurities. Alternatively, pretreatment of the plant material (100 atm at 40 °C for 2 h) without flowing of supercritical CO_2_, followed by extraction with methanol in an ultrasonic bath presented appreciably higher yield than that obtained by UAE without pretreatment (18.4 versus 13.3 mg/g dry plant material, and 2.3 versus 1.6 mg/g dry plant material for hyperforin and adhyperforin, respectively) due to penetration of CO_2_ in the tissue structure that allowed better contact with methanol. The obtained extract contained also hypericin, and more specifically it was recovered in higher yield from the pretreated material, i.e., 1.5 mg/g dry plant material versus 0.8 mg/g dry plant material from the non-treated material [170].

In general, hypericin, flavonoids, and phenolic acids of the plant are quantitatively recovered by more polar solvents (i.e., methanol, ethanol, or aqueous solutions) at elevated temperature. Liu et al. [172] comparing polar and non-polar solvents (water, ethanol, acetone, chloroform, and hexane) found that mixtures of ethanol with acetone were the most effective, and the optimum conditions for the extraction of flavonoids and hypericin were 44–69% ethanol in acetone, for 5.3–5.9 h, at 55 °C, under stirring. However, they mentioned that high temperature increases the extraction yield but leads to hypericin degradation. Similarly, ethanol proved the most efficient in hypericin extraction (yield 1.2 mg/g, dry basis) compared to 2-propanol and ethyl acetate, while n-hexane extracted very low quantity (Soxhlet extraction) [166]. Ethanol was also reported as the best solvent for the efficient quantification of hypericins and their presumed precursors (emodin, skyrin, and skyrin derivatives), while acetone and 80% methanol were also potent extractants [173]. Acetone was more effective for the extraction of hypericins, compared to methanol and tetrahydrofuran, opposed to flavonoids and phenolic acids that were more quantitatively extracted by the latter [161]. In the same study hexane and methylene chloride proved ineffective.

As hypericins were considered the main components providing the pharmacological activities of the plant, several researchers focused on their separation and purification from the crude hydroalcoholic extracts. A liquid–liquid extraction technique followed by preparative column chromatography resulted in a product with 98% purity, while the remaining aqueous extract was rich in flavonoids and could be used as a potent antioxidant [174]. Various chromatographic techniques have been also tested [175].

With regards to the extraction methods, the maximum recovery of hypericin was obtained through digestion or Soxhlet extraction with methanol [157]. Additionally, the flavonoids yield was approximately duplicated by Soxhlet extraction with either methanol or ethanol, compared to UAE or stirring at ambient temperature [157]. Smercerovic et al. [158] compared different extraction methods by using methanol (at solid/liquid 1/20) and found UAE with direct sonication (60 W, 1 h) more effective than Soxhlet extraction (24 h), ASE (40 °C, 100 atm), or maceration (24 h). However, the obtained recoveries of the active compounds were considerably lower than those reported by Avato et al. [157] (Table 5). Different extraction methods were also examined by Williams et al. [161], by using methanol as solvent, and quantifying flavonoids, hypericins but not hyperforins. Soxhlet extraction (24 h) presented better results than indirect UAE (60 °C, 2 h) and the latter better than ASE (60 °C, 152 atm, 0.5 h). However, increase of ASE temperature to 150 °C increased substantially the yield of all components, except quercetin that was probably degraded above 100 °C, while repeated extractions (6–8) with fresh solvent gave results comparable to solvent extraction. The authors commented that longer extraction times and higher temperatures favor the extraction of most components. Another study [160] with 70% ethanol in water at a solid/liquid 1/50, g/mL, concluded that ASE (120 °C, 100–150 atm, 20 min) and MAE (75 °C, 30 min) provided higher recovery (by 20–35%) of the bioactive components compared to extraction with indirect UAE (25 °C, 30 min), and static extraction under heating (solvent boiling point, 90 min). However, decreasing the solid to liquid ratio to 1/100, improved considerably the yield of the static extraction under heating.

The contradictory conclusions, presented above, about the effectiveness of the different extraction methods are probably due to different apparatus and mostly to different temperature, time, and solid/solvent used by each research team. In a study of quercetin (that was the most abundant component) extraction optimization, by using aqueous methanol under indirect UAE, the optimum conditions were determined as methanol concentration 77%, acidified with HCl (1.2 M), extraction temperature 67 °C, extraction time 67 min, and the obtained quercetin yield amounted to 11.1 mg/g dry plant [176]. In the obtained extracts no rutin was detected, possibly because it was hydrolyzed to quercetin under the acidic conditions used. The extraction methods and parameters and the main results are summarized in Table 6. Additionally, a review about the reported extraction methods and the respective compound recoveries is provided by Milevskaya et al. [74]. 

Comparing plants from different districts, it was evident that the consistency varied widely [157]. For example, the content of flavonoids and phenolic acids varied from 13.7 to 35.9 mg/g dry plant, and that of phloroglucinols and naphthodianthrones from 4.6 to 13.4 [160]. The collection period affects the consistency, and the plant collected at the end of the flowering period contains higher quantities of phloroglucinols, while the plant collected at the beginning of the flowering period exhibited higher hypericins content [151,166].

Fractionation of St. John’s wort methanol extract and examination of the antioxidant activity of each fraction by FRAP, DPPH, superoxide, and NO radicals scavenging assays, indicated that the antioxidant activity was mostly attributed to flavonoid glycosides and phenolic acids (chlorogenic acid), while biflavonoids (lacking the catechol moiety), naphtodianthrones, and phloroglucinols showed very low activity [178]. Kalogeropoulos et al. [179] reported quercetin glycosides (hyperoside, quercitrin, and isoquercitrin) and catechin as the main flavonoid constituents of the methanol extract and associated them with the observed DPPH radical scavenging activity. The ethanol in water (80%) extract of the plant presented activity against several radicals and also a high capacity to inhibit iron-mediated lipid peroxidation [180]. Correlating this capacity with the flavonoid profile of the plant and the antiradical activities of the identified components, the authors suggested that it should be attributed to the presence of components that possess antiradical properties together with iron-binding ability (mainly quercetin and kaempferol, but also biapigenin and quercetin glycosides). Additionally, 10% aqueous glycerol solutions and water infusions were found rich in chlorogenic acid and quercetin glycosides—but did not contain either hypericin or hyperforin—and presented appreciable FRAP, DPPH radical scavenging, and acetylcholisterenase inhibition activity [177,181].

## 7. Saffron

*Crocus sativus* L. (saffron) is a perennial herb belonging to Iridaceae family, originated and evolved in Attica (Greece) from the wild *Crocus cartwrightianus*, and probably domesticated there [182,183]. Since the Bronze Age saffron was grown in the eastern Mediterranean territories and the Middle East, while currently the cultivation of the plant has spread to more territories such as the USA, China, and Australia. However, the main production areas include a geographic zone between the Mediterranean, the Middle East countries, and India. Iran has been traditionally the main producer, accounting for more than 90% of the world production, followed by far by India, Greece, and Spain. The valuable part of the plant are the stigmas of flowers, with the requirement of harvesting 150,000–200,000 flowers by hand, to get 1 kg of saffron stigmas [183]. This extremely low yield renders the saffron stigmas as the most expensive spice of the world.

The composition of stigmas, concerning the secondary metabolites, includes a major group of glycosylated apocarotenoids, named crocins, which are responsible for the dark red color of stigmas. The respective carotenoid aglycone of crocins is the C_20_ dicarboxylic acid crocetin (8,8′-diapocarotene-8,8′-dioic acid), and all of them are water soluble components [184]. The C_20_ apocarotenoids of saffron could be distinguished to the all-*trans* members, including crocins and crocetin, and the 13-*cis* members, which are exclusively crocins (Figure 1b). However, the typical composition of the water-soluble apocarotenoids of saffron include two major all-*trans* compounds, namely *trans*-crocetin di-(β-d-gentiobiosyl) ester and *trans*-crocetin (β-d-gentiobiosyl)-(β-d-glucosyl) ester, followed by the minor *cis*-crocetin (β-d-gentiobiosyl)–(β-d-glucosyl) ester, while all of the other, approximately 13 components occurring in water extracts of saffron, appear as even minor components or traces, including crocetin [184,185].

The abbreviations of crocins followed by most researchers can be detected as early as 1995 at the manuscript of Tarantilis et al. [186]. At the respective paper, authors abbreviate each crocin as crocin-n, where n indicates the total number of glucose moieties. Therefore, according to Tarantilis et al. [186] the major crocin (*trans*-crocetin di-(β-d-gentiobiosyl) ester) is abbreviated as crocin-4, while *trans*-crocetin (β-d-gentiobiosyl)-(β-d-glucosyl) ester as crocin-3. Crocins-2 are discriminated as crocin-2 [*trans*-crocetin β-d-gentiobiosyl ester] and crocin-2′[*trans*-crocetin di-(β-d-glucosyl) ester]. Carmona et al. [187] extended the abbreviations so as to present more structural details, i.e., the first part describes the *cis*/*trans* form of the aglycone, followed by the total number of sugar moieties (glucose monomers), and finally, the type of sugar in each part of the crocin structure (G refers to gentiobiose; g, to glucose; n, to neapolitanoside; t, to triglucoside). Therefore crocin-4 is abbreviated as *trans*-4-GG, according to Carmona et al. [187], while crocin-3 as *trans*-3-Gg. Synthesizing the two systems, *trans*-crocetin di-(β-d-gentiobiosyl) ester should be abbreviated as *trans*-crocin-4-GG or *trans*-4-GG crocetin ester, however the versions of *trans*-crocin-4 or *trans*-crocin 4 are mainly detected in the literature, assuming that the glycosylation pattern of the compound is generally taken for granted. Other types of abbreviations such as the one of Siracusa et al. [184] are used in a far lesser extent in the literature. Crocins are coded as crocin-n, where n is, assumingly, an indication of the abundance of the respective compound. The most abundant *trans*-crocetin di-(β-d-gentiobiosyl) ester is named as crocin-1, the second *trans*-crocetin (β-d-gentiobiosyl)-(β-d-glucosyl) ester as crocin-2 etc.

Other significant components of saffron stigmas are the C_10_ picrocrocin (a bitter taste glucoside) and safranal (Figure 1b), the main volatile component of saffron, responsible for its particular aroma, both of which derive from the carotenoid oxidation pathway [184].

Apart from the value of stigmas as spice, they appear to be a significant source of bioactive components against a broad range of pathological conditions and disorders as presented in numerous reports. In recent papers it has been evidenced that crocins can modulate the serum lipid profile in patients with metabolic disorders [188], prevent cancer, and present antitumor activities, according to experiments with cultured human malignant cell lines and animal models [189]. Kazemi et al. [190] have demonstrated that treatment of patients, with mild to moderate obsessive-compulsive disorder, with saffron crocin is equally effective to fluoxetine, while saffron intake has been associated with improvements in sleep quality in adults with self-reported sleep complaints [191].

The potential valorization of crocins, picrocrocin or safranal as novel natural pharmaceuticals or even nutraceuticals require efficient extraction protocols. Thus, various research teams focused on the development of extraction methods. It is noted that several researchers have been based on the recommended extraction protocols of the ISO, such as the ISO 3632-2:2010 [192], and tried to optimize them with the use of assisting techniques, i.e., UAE or MAE. In the above protocol the solid-to-liquid ratio equals 1/2000 and ensures practically the total extraction of bioactives. The optimization of such procedure could be applicable only for analytical purposes, due to the high dilution of the extract. The potential valorization of an extract requires the minimum use of solvent with the highest yield of compounds.

Table 7 presents the research works that go beyond the analytical purposes and approach the production of saffron extracts promising for industrial application. The basic extraction protocol of saffron includes water as extraction solvent, since the major components, crocins and crocetin, are water-soluble. However, several pure organic solvents such as ethanol, methanol, acetone, etc., have been applied [193] with limited success.

Mixtures of various organic solvents with water have also been examined, with ethanol–water and methanol–water to surpass other systems [194,195,196,197,198]. The references dealing with ethanol–water or methanol–water mixtures could be distinguished in two categories; those that implement conventional extractions methods, i.e., agitation and maceration [194,195] and the ones that apply the modern assisting techniques of UAE and MAE [196,197,198]. CSEs applied ethanol–water 1:1 mixtures, with high solid-to-liquid ratios, namely 1/20 and 1/30 [195,196]. Montalvo-Hernandez et al. [194] succeeded a 77% recovery of saffron crocins agitating at 200 rpm for 1 h a mixture of saffron powder and ethanol–water (50%) at ratio of 1/20. The use of assisting techniques, significantly reduced the duration of extractions that ranged between 5 and 30 min [197,198,199]. Especially Kyriakoudi et al. [197] with 29 min extraction time, methanol 50% in water, at solid/liquid ratio 1/180, under UAE, reached a total yield of 627 mg crocetin esters/g saffron, which is the highest value found in the literature. High hydrostatic pressure (HHP) can be considered a novel assisting technique of extraction; Shinwari et al. [199] applied HHP to a mixture of saffron powder with water (solid/liquid, 1/100), pressure 5800 atm, temperature 50 °C, time 5 min, and recovered crocins (yield amounting to 25% of theoretical) and picrocrocin.

SFE has also been applied to recover saffron components. Pure CO_2_ has been used for the extraction of safranal and non-volatile lipids [200,201,202], while concerning crocins, picrocrocin and crocetin the use of a modifier was considered necessary [202,203]. Nerome et al. [203] succeeded the recovery of crocin and picrocrocin, at respective yields of 68.8% and 88.4%, with the use of water as a modifier.

While stigmas are considered as the valuable part of the herb, the petals amount to 99.7% of harvested material and remain unexploited. According to Caser et al. [204] 1 kg of stigmas correspond to 350 kg of petals, which are regarded as agricultural waste. However, in recent years, and in the frame of new ecofriendly trends such as the circular economy and the sustainable growth, the valorization of petals has gained the interest of the scientific community. According to early studies, saffron petals contain flavonoids with major components the glycosylated derivatives of quercetin and kaempferol, including their methoxylated and acetylated derivatives [204,205]. In the study of Termentzi and Kokkalou [205], the CSE of saffron petals with methanol yielded 12% *w/w* in extractable components. Authors determined the flavonoid concentration in the methanolic extract 13%, thus the flavonoid content reaches 16 mg/g on petals basis. Further analyses revealed a total alkaloid yield in the magnitude of 0.9 mg/g, and the monoterpene crocusatin at 57.5 ppm. Furthermore, five anthocyanins have been reported (delphinidin, petunidin, and malvidin glycosides) and quantified at 4.8 mg/g, and lutein diesters (215 ppm, on petals basis) [206].

The studies on the application of extraction methods at larger than the analytical scale have started recently, since 2016, and include conventional extraction methods, such as maceration, and novel techniques. In most cases conventional methods have been used for comparison reasons with modern assisting techniques, i.e., UAE, MAE, Ohmic heating assisted extraction (OHAE), SFE, and SWE. Water, ethanol–water, and CO_2_–ethanol mixtures appeared until recently as the only solvents used for the recovery of compounds. In a research paper of 2021 [207], methanol was used for the first time, in mixtures with water, as potential solvent for petals extraction. The extraction methods from the literature together with their respective optimization parameters are summarized in Table 8.

The first study of petals extraction with potential application in large scale is the one of Ahmadian-Kouchaksaraie et al. [208], who optimized the SWE of petals powder. They concluded that the optimal conditions for SWE were solid/liquid 1/36, temperature of 159 °C, and time 54 min. At the above conditions they achieved a total phenol yield of 16.2 mg/g dry plant powder, and flavonols yield of 2.39 mg/g. Water was also used by Hashemi et al. (2020) [213]. They performed CSE (agitation) of petals with hot water (66 °C) at a solid/liquid ratio of 1/36, for 104 min and yielded TPC 7.21 mg/g, TFC 1.01 mg/g, and TAC 1.89 mg/g. Additionally, Stelluti et al. [207] macerated petals powder with water under stirring (1000 rpm) in the dark for 30 min, at 21 °C, yielding TPC 11.4 mg/g and TAC 3.45 mg/g.

Khazaei et al. [209] optimized the extraction of anthocyanins, macerating saffron petals with hydroalcoholic mixtures; at the optimal conditions (solid/liquid 1/20, ethanol concentration in water 25%, extraction temperature 25.8 °C, duration of extraction 24 h) they obtained 32 mg total monomeric anthocyanins/g dry material. The maximum yield of anthocyanins detected in the literature, reached 101 mg/g dry tepals powder by Jafari et al. [212]. The researchers obtained such a high yield by extracting petals with a mixture of acidified ethanol–water at solid/liquid ratio 1/20 using MAE. In all other cases the total anthocyanins yield, ranged between 1 and 4 mg/g.

Ahmadian-Kouchaksaraie and Niazmand [210] optimized SFE of petals. They used a volume of distilled preheated water before filling the vessel. For extraction by SC CO_2_, the ratio of solid-to-liquid ratio was 1/30 with 5% (*v/v*) of ethanol, while the optimal conditions were determined 62 °C, 47 min extraction time, and pressure of 164 atm. The yield of TPC reached 14.2 mg/g, total flavonoid content (TFC) 1.8 mg/g, and total anthocyanin content (TAC) 1 mg/g.

As far as the assisting techniques are concerned, we can notice that solid/liquid ratios ranged between 1/50 and 1/20, and durations 2–45 min [207,211,213]. Compared with the respective conventional extractions the TPC yields varied between 96 and 129% of the theoretical values. The MAE method proposed by Hashemi et al. [213] could be distinguished, since they used the highest solid to liquid ratio, namely 1/20, water (with 0.3% NaCl content) as solvent, and increased the yield of TPC by 21%, in comparison with the respective conventional method, applying a rapid extraction protocol, that lasted only 4.25 min.

In general, crocins are recognized as the most valuable bioactive components of saffron. Assimopoulou et al. [214] studied their antiradical activities against DPPH and especially the activity of crocin-4. Using a DPPH protocol with 2 h incubation, they determined EC_50_ = 0.516 mg_crocin_/mg_DPPH_, a value that can be converted to 0.21 mol_crocin_/mol_DPPH_, according to the respective M_r_ values of crocin and DPPH. Therefore, the number of radicals scavenged by each crocin molecule (*n*) corresponds to 2.4 (1:2.4) based on the formula *n* = 1/(2 × EC_50_), where EC_50_ is expressed as mol_crocin_/mol_DPPH_. A stoichiometric factor of such magnitude is close to the respective values of potent antiradical structures, such as the B-catecholic flavonoids [215,216]. Dar et al. [217] determined even higher activity, comparing crocin with kaempferol (*n =* 2, [216]) and ascorbic acid, that in terms of IC_50_ (expressed as µmol/mL) the two latter present similar activity [217].

Apart from extracting crocins, further efforts have been held in order to formulate stable extracts, in which the bioactive components are protected from environmental conditions, and thus could be easily handled as new ingredients in foods, cosmetics, and nutraceuticals. The encapsulation of crocins in protective matrices such as maltodextrin and alginates with further spray and freeze drying have already been successfully applied [218,219,220], and increase the possibility of the commercial use of saffron extracts. Ensuring the protection of crocins with appropriate carriers, then the respective commercial products could be standardized in terms of crocin content, probably according to the ISO protocols.

## 8. Conclusions

*R. officinalis*, *O. vulgare*, *M. officinalis*, and *S. thymbra* are among the most potent members of the Lamiaceae family for the extraction of antioxidant compounds. All of them contain appreciable amounts of phenolic acids (mainly rosmarinic acid) and flavonoids, which have been associated with the antioxidant activity and several health-beneficial effects of their extracts. Extracts rich in antioxidant compounds can be obtained by ethanol in water solutions (around 60%) and the extraction time may be shortened by a moderate increase of temperature (up to 60 °C) or by UAE application. Water can be also used, although the recovery (especially of the flavonoid aglycones) and the purity of the extracts is lower. ASE is a promising technique that enhances mass transfer phenomena and reduces extraction time, provided the necessary equipment is available. Pure ethanol applied in ASE or UAE presented high yield and selectivity for phenolic compounds, thus providing extracts with high antioxidant activity.

Both UAE and ASE have emerged as fast extraction techniques that need lower amounts of solvents, and therefore are more economical and have a lower environmental impact. Moreover, the use of ethanol and mostly water renders them green extraction techniques. In particular in ASE applications, the use of high temperature decreases the dielectric constant of water providing properties similar to organic solvents. Thus, water can be a real alternative to organic solvents in applications that do not involve the extraction of compounds with high temperature sensitivity. MAE has similar advantages to UAE and ASE but the high rate of microwave energy absorbance by water may result to overheating and destruction of sensitive compounds, thereby the use of mixtures with organic solvents seems necessary to obtain good yields.

In addition to phenolic acids and flavonoids, *R. officinallis* contains phenolic diterpenes (carnosic acid and carnosol) that exhibit high antioxidant activity, especially in lipid substances. Acetone or hexane, with the aid of UAE, can be used to extract selectively the phenolic diterpenes and obtain high purity of the extracts. Nevertheless, acetone in water, or ethanol in water (60–80%) lead to high recovery of all antioxidant components (carnosic acid, carnosol, rosmarinic acid, and flavonoids).

*H. perforatum* contains several flavonoids and phenolic acids but, also, phloroglucinols and naphtodianthrones that have several medical applications. Pure extracts of phloroglucinols are difficult to achieve, and can be obtained by hexane extraction or SFE with neat CO_2_. On the other hand, naphtodianthrones cannot be separated from flavonoids and extraction procedures with ethanol in water solutions provide the best results for a simultaneous recovery of all compounds, similarly to the Lamiaceae family plants.

*Crocus sativus* is a rich source of bioactives, both from stigmas and petals. The most valuable compounds of stigmas include crocetin glycosides, which can be efficiently extracted either with water, water/ethanol, or water/methanol mixtures. The assisting techniques significantly reduce the duration of extraction and increase both solid/liquid ratio and yield, especially for the latter reaching 627 mg/g saffron. The petals of saffron that are considered as a byproduct of stigmas production, contain methoxylated and acetylated flavonol aglycones and glycosides, and anthocyanins. The content of the above compounds, ranges in a much lower level than the respective of crocins in stigmas however in recent years, many researchers have attempted the valorization of petals. Novel assisting extraction techniques have been employed, achieving very short durations of extractions, e.g., 2–10 min, with the use of water in high solid/liquid ratios, such as 1/20, or ethanol–water mixtures, and in combination with the abundance of the raw material could lead to industrial applications.

Future research should focus on the scale up of extraction techniques to allow industrial application. With regards to CSE, semicontinuous or continuous extraction procedures must be examined as they allow solvent recycling and more feasible production costs. For the novel techniques (UAE, ASE, and MAE), the degradation kinetics of the sensitive compounds should be clearly defined, so as to optimize the extraction parameters, i.e., extraction time and temperature. Additionally, extensive research is carried nowadays about the health effects of several phenolic compounds, like rosmarinic acid. The results of this research may guide the future efforts towards the selective extraction of the specific health-promoting compounds and standardization of the relevant extracts.

## Figures and Tables

**Table 1:** Solvents and methods reported in literature for the extraction of phenolic compounds from rosemary.

Solvent	Method	Measured Parameters	Main Results	Reference
Butanone Ethyl acetate Ethanol (solid/liquid 1/10, *w/v*)	CSE (25–50 °C, 0.25–3 h) UAE (probe 20 kHz) UAE (bath 40 kHz)	CA	CA yield increased with temperature. UAE probe or bath gave similar results and decreased extraction time (0.25 h compared to 3 h at 50 °C by CSE to obtain 15 mg CA/g dry plant)	[24]
Ethanol Methanol (solid/liquid 1/20, *w/v*)	CSE UAE (probe 20 kHz) UAE (bath 40 kHz) 25–50 °C, 0.25–2.0 h	CA RA DPPH	Ethanol gave higher yield of CA and methanol of RA and antiradical activity. UAE leads to more effective extraction, at lower temperature with less dependence on solvent Scale up (125 L) with ethanol resulted in 22 and 1.6 mg/g dry plant for CA and RA, respectively.	[26]
Hexane Acetone Ethanol Water (solid/liquid 1/10, *w/v*)	UAE (probe 20 kHz, 10 min) MAE (under N_2_, 100 °C, 10 min) UAE: Single or successive extractions	HPLC	UAE with ethanol or acetone gave the highest terpenoids yield. Highest TPC was obtained with UAE or MAE with ethanol (35 and 36 mg/g dry plant, respectively). UAE with hexane showed a high selectivity in CA extraction, and with acetone low CA oxidation	[25]
Ethanol Methanol Acetone Water mixtures	CSE (ethanol in water 44.8–95.2%, solid/liquid 1/4.6–1/21.4, *m*/*v*, time 4.8–55.2 min)	CA COH RA	Ethanol 59% or 70% and acetone 80% gave the best results for all three compounds. Optimum conditions: ethanol 70%, solid/liquid 1/5, extraction time 55 min to obtain highest yield and antioxidant concentration in the extract	[22]
Ethanol in water (0–96%) Acetone in water (0–100%) (solid/liquid 1/20, *w/v*)	CSE UAE Pretreatment: deoiling by water-steam distillation, milling, maceration	TPC HPLC DPPH	60% ethanol or acetone showed the highest TPC yield and concentration in the extract. Highest RA yield was obtained with water gave, flavonoids with 60% acetone, and terpenes with 80% acetone UAE enhanced TPC extraction and antiradical capacity of the extract, especially with ethanol 60%. Grinding increased the extraction rate.	[21]
Ethanol water (solid/liquid 1/6, *w/v*)	CSE (40 °C, 4 h) UAE (probe) MAE Pretreatment: deoiling by solvent free MAE, milling	Yield TPC CA RA DPPH	CA not detected in water extracts. Higher yields of TPC, RA and lower EC_50_ in water extracts. UAE and MAE decreased extraction time. De-oiling and milling increased yield, TPC and RA content in the extract.	[23]
Ethanol in water Water	CSE (solid/liquid 1/10–1/20, *m*/*v*, 27–70 °C, 30–300 min UAE probe (solid/liquid 1/20, *m*/*v*, 40–90% ethanol in water, 40 °C, 60–200 W, 3–13 min)	Yield TPC DPPH	30 min by CSE or 11 min for UAE were sufficient to obtain the maximum TPC and antiradical efficiency. Combination of CSE (step 1) and UAE (step 2) did not improve results. 56% ethanol presented best results in either CSE or UAE	[31]
Ethanol in water 70%, 90% Water + Tween 20 (solid/liquid 1/15, *w/v*)	UAE bath Maceration (90% ethanol, room temperature, 48 h) Percolation	TPC RA UA OA DPPH	The highest yield of UA (15.8 mg/g) was obtained by UAE with 90% ethanol, 60 °C, 10 min; RA (15.4 mg/g) by UAE with 70% ethanol, 50 °C, 30 min, or water (at pH 9); and OA (12.2 mg/g) by maceration. Highest TPC was obtained by water extraction.	[18]
Ethanol in water 90% (solid/liquid 1/20, *w/v*)	Heat reflux extraction (78 °C, 0.5 or 5 h) Maceration (40 °C, 0.5 h) UAE bath/reactor/probe (40 °C, 0.5 h) MAE under reflux (78 °C, 0.5 h) under N_2_ pressure under vapor pressure	RA CA UA COH	Heat reflux extraction for 0.5 h resulted in extraction yield of 19%, compared to 10% obtained by maceration. UAE with probe showed similar yield to heat reflux extraction but higher recovery of CA and UA. In MAE, extraction and RA yields increased with temperature but CA and UA yield decreased. Pressure does not enhance extraction.	[29]
Ethanol in water 30–96% (solid/liquid 1/5, *w/v*)	Maceration (3 days with occasional shaking) Percolation	TPC DPPH	Highest TPC obtained with 50%, no significant differences in antiradical activity Percolation gave higher TPC yield but lower antiradical activity.	[28]
Water Methanol:water (60:40) Acetone:water (60:40) Ethyl acetate:water (60:30) (solid/liquid 1/40, *w/v*, 1/20 in MAE)	MAE (4 min, under N_2_) Heat reflux extraction (90 °C, 2 h, under N_2_)	TPC HPLC	MAE gave comparable TPC yield to conventional extraction at shorter time Acetone in water presented highest TPC yield in MAE. Water presented the highest TPC in heat reflux extraction followed by methanol, acetone and ethyl acetate in water mixtures. Content of individual phenolics was similar in either method	[32]
Methanol:water 50:50–100:0 Ethanol:water (70:30) Acetone:water (70:30) Ethyl acetate:water (70:30) (solid/liquid 1/5, *w/v*)	MAE (2 × 1–2 × 15 min) UAE bath (2 × 5 min) Soxhlet (1–5 h)	TPC flavonoids, anthocyanins	MAE gave comparable TPC yield with the optimum obtained in Soxhlet extraction (3 h), and 2-fold higher than UAE. Maximum TPC with methanol:water, 70:30, flavonoids with ethanol:water, 70:30, anthocyanins ethanol:water, 70:30 + 1% HCl, for 2 × 5 min.	[33]
Methanol in water 32–88%	Maceration (1/50, *w/v*, 80% methanol, room temperature, overnight) ASE (66–200 °C, 103 atm)	TPC, HPLC FRAP	Optimum conditions through RSM: 56% methanol, 129 °C. TPC (101.7 mg/g dry herb) and antioxidant recovery at optimum ASE conditions were higher than those obtained by solid/liquid extraction.	[30]
Ethanol Water (solid/liquid 1/10, *w/v*) CO_2_ CO_2_ + 7% ethanol	ASE (50–200 °C, 100 bar, 20 min) SFE (40 °C, 100–400 bar, 300 min) WEPO	TPC DPPH HPLC	ASE with water gave the highest yield and antioxidant activity of the extract. TPC, yield and antiradical activity increased with temperature and water was more efficient than ethanol. The extract obtained by SFE with CO_2_ + 7% ethanol had good TPC and antiradical activity but low yield.	[34]
Ethanol Water (solid/liquid 1/10, *w/v*) CO_2_ CO_2_ + 6.6% ethanol	ASE (150 °C with ethanol, 100 or 200 °C with water, 100 bar, 20 min) SFE (40 °C, 150, 400 bar)	HPLC	SFE extracted compounds of low polarity. RA was extracted by ASE with either solvent, while most flavonoid glycosides were extracted only by ASE with water	[19]
Ionic liquids in water (solid/liquid 1/20, *w/v*)	UAE (bath 100–250 W, 0.5 h, after 2 h soaking)	CA RA	The extraction efficiency was comparable to 80% ethanol used in UAE (0.5 h), solvent extraction (24 h) or CSE (24 h).	[35]

ASE: accelerated solvent extraction, CA: carnosic acid, COH: carnosol, CSE: conventional solvent extraction, DPPH: 1,1-diphenyl-2-picrylhydrazyl radical, FRAP: ferric reducing antioxidant power, MAE: microwave assisted extraction, OA: oleanolic acid, RA: rosmarinic acid, SFE: supercritical fluid extraction, TPC: total phenolic content, UA: ursolic acid, UAE: ultrasound assisted extraction, WEPO: pressurized water extraction with particle on-line formation.

**Table 2:** Solvents and methods reported in literature for the extraction of phenolic compounds from oregano.

Solvent	Extraction Method/Parameters	Measured Parameters	Main Results	Reference
Ethanol, Supercritical CO_2_ (Ethanol as modifier)	Focused UAE (20 kHz, 10 min) SFE (CO_2_ flow: 1–2 mL/min, T: 35–60 °C, P: 100–170 atm, ethanol 5–40%)	TPC TEAC (ABTS)	Optimum conditions determined by experimental design: Focused UAE (50% amplitude, 12.5 min) SFE (CO_2_ flow: 1 mL/min, T: 40 °C, P: 100 atm, 40% ethanol) TPC almost twice extracted by means of the Focused UAE.	[85]
Supercritical CO_2_ (Ethanol as modifier)	SFE (40–60 °C, 150–350 atm, fractionation of EO and phenolic compounds)	HPLC DPPH *β*-carotene-linoleic acid	Extraction yield increased with ethanol. Higher antioxidant capacity at 40 °C, 250 atm. Dihydroquercetin only extracted with ethanol as modifier.	[86]
Water	ASE (25, 50, 100, 150, 200 °C, 103 atm, 30 min-individual extractions, or sequential extractions for 15 min at each temperature)	TPC DPPH HPLC	Extraction yield was higher at individual extractions, and increased with temperature. TPC of the extracts was not affected by temperature, but antioxidant activity Increased.	[84]
Ethanol in water (0–96%) (s/l 1/40–1/20, *w/v*)	CSE Temperature (22–60 °C), particle size (<315–1000 μm)	TPC DPPH HPLC	Optimum conditions: 60% ethanol, s/l 1/20, 22 °C, 600 μm. Increase of ethanol up to 60% increased RA and lithospermic acid yield.	[64]
Methanol in water (70–90%) (s/l 1/20–1/5, *w/v*)	CSE (Extraction time 4, 10, 16 h, particle size 20, 65, 110 μm)	TPC DPPH	All examined parameters were significant. Optimum conditions (RSM): MeOH (70%), s/l 1/20, time 16 h, particle size 20 μm TPC (18.75 mg/g dry herb)	[78]
Methanol in water (32–80%)	Maceration (s/l 1/50, *w/v*, 80% methanol, overnight) ASE (66–200 °C, 103 atm)	TPC FRAP HPLC	Optimum ASE conditions (RSM): 33% methanol, 129 °C. TPC and FRAP at optimum ASE conditions were higher than those obtained by maceration. RA and luteolin-7-*O*-glucoside showed a decrease at T ≥ 150 °C in comparison to optimum ASE.	[30]
Ethanol in water 90%, *v/v* s/l 1/100 Ethanol in propylene glycol (10–30%) Ethanol in glycerol (1–20%)	CSE (25 °C, 24 h) Heat-reflux (95 °C, 1–6 h) Maceration (s/l 1/100, 24 h or 1/5, 48 h) Percolation (25 °C, 48 h) UAE (Ethanol 30–96%, s/l 1/20, 25–60 °C, 10 min)	RA UA OA CAR	Heat reflux and CSE gave the highest yields, while percolation the lowest. During heat reflux: Ethanol in non-aqueous solvents was more effective. Highest RA: Heat reflux (Ethanol, 90% *v/v*, 6 h) Highest OA, UA: Maceration (Ethanol, 90% *v/v*, 1/5, 48 h) Highest CAR: UAE (Ethanol, 96% *v/v*, 25 °C) or Continuous stirring	[65]
Methanol in water 80% *v/v* Water	CSE (25 °C, 60 min, 150 rpm) Infusion (5 min) Decoction (boiling in water, 5 min)	HPLC DPPH *β*-carotene-linoleic acid lipid peroxidation	CSE extracts showed higher antimicrobial activity. Higher TPC, flavonoids and antioxidant capacity by decostion > infusion > CSE	[69]
Water, Ethanol, Acetone, Ethyl acetate, Diethyl ether	Maceration (Three times, 25 °C, 24 h)	TPC Flavonoids Condensed tannins DPPH	Highest TPC: water Highest flavonoids: Diethyl ether Highest Condensed tannins: Diethyl ether Highest antioxidant capacity: Ethanol	[70]
Methanol in water 70%, *v/v* Water	UAE (bath) (T < 30 °C, 20 min)	DPPH FRAP ABTS HPLC	Hydroalcoholic extract contained higher RA, CAR and TPC.	[79]
Methanol in water 80%, *v/v*	UAE (12.5 kHz, 30 min)	TPC HPLC ORAC	There is a correlation between ORAC and TPC, but not between ORAC and RA.	[80]
Water (s/l 1/100, *w/v*)	Infusion (85 °C, 15 min) Infusion (25 °C, 15 min) UAE (35 MHz, 25 °C, 15 min)	TPC ABTS DPPH	Hot water showed the highest efficiency for oregano and lemon balm. Lemon balm had higher TPC than oregano	[87]

ABTS: 2,2-azino-bis-3-ethylbenzothiazoline-6-sulfonic acid radical, ASE: accelerated solvent extraction, CAR: carvacrol, CSE: conventional solvent extraction, DPPH: 1,1-diphenyl-2-picrylhydrazyl radical, FRAP: ferric reducing antioxidant power, OA: oleanolic acid, RA: rosmarinic acid, SFE: supercritical fluid extraction, TEAC: trolox equivalent antioxidant capacity, TPC: total phenolic content, UA: ursolic acid, UAE: ultrasound assisted extraction.

**Table 3:** Extraction techniques and main results reported in literature for the recovery of phenolic compounds from pink savory.

Solvent	Method	Measured Parameters	Main Results	Reference
Ethyl acetate Ethanol (solid/liquid 1/10, *w/v*)	Soxhlet successive extraction (6–8 h, until the extract was colorless) Pretreatment: water-steam EO distillation	TPC DPPH Antioxidant activity in palm oil and oil-in-water emulsions	The TPC content followed the order ethanol extract > aqueous extract from EO distillation > ethyl acetate extract. Ethanol and aqueous extracts exhibited good antiradical activity, ethyl acetate a moderate one. Ethyl acetate extract showed antioxidant activity in palm oil, and ethanol extract in emulsions.	[101]
Aqueous solution of potassium hydroxide (KOH), 1%, 3%, and 5% (*w/v*)	Maceration with stirring (room temperature, 0.5, 3, 6, and 24 h) Pretreatment: water-steam EO distillation	TPC DPPH Oxidative stability index	High TPC and good antiradical and antioxidant activity of the extracts in 30 min of extraction with KOH 1% (*w/v*). The increase of extraction time and KOH concentration caused lower TPC, antioxidant, and DPPH radical scavenging ability of the extracts.	[2]
Methanol in water 70% of Water	UAE (bath, less than 30 °C, 20 min)	HPLC DPPH FRAP ABTS TPC	A reversed phase HPLC method has been developed for the determination of 24 phenolic compounds in five aromatic plants of the Lamiaceae family. Methanol 70% was more effective than water.	[79]
Methanol (solid/liquid 1/33.3, *w/v*)	Heat reflux extraction (water bath, 1 h)	HPLC	Isolation, qualification, and quantification of free phenolic acids in plant material. Removal of interfering compounds (chlorophyll, waxes, and polyphenols) by means of a solid phase extraction clean-up on an octadecyl sorbent and anion exchange resin.	[105]
Glycerol-based ionic liquids	Maceration with stirring (600 rpm), at 50 °C for 200 min	HPLC TPC DPPH FRAP	Optimum water concentration 54.8–63.8% (*v/v*) and s/l 1/30–1/36, *w/v*. LTTMs displayed anti-Arrhenius kinetics over a temperature ranging from 40 to 80 °C, evidencing peculiar extraction behavior.	[106]

ABTS: 2,2-azino-bis-3-ethylbenzothiazoline-6-sulfonic acid radical, DPPH: 1,1-diphenyl-2-picrylhydrazyl radical, EO: essential oil, FRAP: ferric reducing antioxidant power, TPC: total phenolic content, UAE: ultrasound assisted extraction.

**Table 4:** Techniques and main results reported in literature for the extraction of phenolic compounds from lemon balm.

Solvent	Method	Measured Parameters	Main Results	Reference
Ethanol (Solid/liquid 1/4–1/10, *w/v*)	CSE temperature 0–80 °C, particle size 200–250, 250–315, 315–400 μm	CA UA OA Extraction yield	Extraction was governed by internal mass transfer (diffusion coefficients according to Fick’s 2nd low were determined) Yield increased with decreasing particle size and solid-to-liquid ratio.	[130]
Ethanol, methanol, acetone or acetonitrile, all 30% in water Water Ethanol 15–96% Solid/liquid 1/500, *w/v*	UAE 10 min	RA CAF	All 30% mixtures showed similar RA recovery 20% higher than pure water. 30 and 60% ethanol in water showed the highest recovery of both acids.	[131]
Methanol in water 40–80% Solid/liquid 1/20, *w/v*	CSE (25–55 °C, extraction time 30–90 min)	RA	The optimum conditions determined by RSM were methanol concentration 59%, *v/v*, at 55 °C, for 65 min and gave RA yield 4.6% on dry leaves.	[132]
Methanol in water Methanol in water (pH 2.5) Ethanol in water Ethanol on water (pH 2.5) Water Solid/liquid 1/50, 1/100, 1/150, *w/v*	CSE, UAE (bath) (25 °C, 5–20 min)	RA CAF ProtCa	UAE was more effective than CSE at the same time. RA extraction was slightly higher with acidified mixtures. Methanol 60%, at a solid-to-liquid ratio 1:100, by 3 successive extractions of 10 min each, recovered quantitatively all phenolic acids.	[119]
Methanol in water 0–100% Ethanol in water 0–100% Acidification with 0.1–1.0% HCl Solid/liquid 1/40, *w/v*	Maceration under stirring (30–1140 min) CSE at boiling point (15–60 min) MAE (50 or 80 °C, 5–30 min)	RA	MAE gave similar results to conventional methods at shorter time (5 min). More than 5 min in MAE and 15 in CSE caused degradation. Ethanol:water:HCl 70:29:1, *v/v/v* gave the best results	[133]
Ethanol in water 20–80%	CSE (25–60 °C)	RA	Highest RA yield at 50% ethanol in water. Increase of temperature caused minor increase in yield that was not significant above 50 °C	[134]
Ethanol in water 80% or 50% Solid/liquid 1/10, *w/v*	CSE of untreated or SFE treated material (40 °C, extraction time 110 min)	RA Extraction yield	Ethanol 50% achieved higher RA yield than 80%. 3 successive extractions were needed to recover RA absorbed in the wet material. SFE pretreatment increased extraction rate and final yield	[120]
Ethanol in water 0–100% Solid/liquid 1/33, *w/v*	CSE (30–90 °C, 30–90 min) UAE (probe 100–500 W, 3–45 min at constant temp.: 30–35 °C) MAE (60–180 °C, 3–45 min)	RA	RSM analysis showed that all studied variables were significant in all methods. UAE gave the highest RA yield (86 mg RA/g dry plan) under the optimum conditions (40% ethanol, 371 W, 33 min).	[135]
Ethanol in water 0, 70, 100% Solid/liquid 1/10, *w/v* in MAE Successive extractions with hexane, acetone, ethanol, water in UAE	UAE (probe 20 kHz, 10 min) MAE (under N_2_, 100 °C, 10 min) Dry extraction by grinding with *β*-cyclodextrin (1:2, *w/w*)	HPLC Extraction yield	MAE with water showed the highest extraction yield, but with 100% ethanol the highest phenolic and RA recovery was shown. UAE was less efficient and the best phenolic and RA recovery was obtained with ethanol and was enhanced when acetone was used in a previous extraction. Dry extraction was the least efficient.	[136]
Ethanol in water 70%, 96% Solid/liquid 1/10, *w/v*	MAE (5–15 min, 25, 40, 60 °C)	TPC	70% ethanol at 10 min and 60 °C showed the highest TPC recovery	[137]
Ethanol Water Solid/liquid 1/20, *w/v*	EAE (cellulose, *β*-xylanase, pectinase, 50 °C, 2 h, pH 5) ASE (150 °C, 20 min)	TPC LC-MS/MS DPPH TEAC (ABTS)	ASE showed the highest TPC yield and antioxidant activity with water being more effective than ethanol. EAE with a combination of all enzymes gave better results than non-enzymatic extraction (pH 5).	[127]
Water Solid/liquid 1/20, *w/v*	CSE (40–100 °C, 5–120 min)	TPC DPPH ABTS	Optimization by RSM. Temperature and temperature-time interaction were significant. Optimum results at 100 °C for 120 min	[138]
Water Solid/liquid 1/10, 1/20, 1/30, *w/v*	CSE (97 °C, 5–30 min) UAE (probe at 150 or 240 W, 5–30 min, and constant temp 40 °C) MAE (97 °C, 5–20 min) Maceration (40 °C, 24 h)	TPC	Phenolics recovery increased as solid-to-liquid decreased. MAE showed the highest TPC recovery (146 mg GAE/g dry plant, at 5 min) and UAE the lowest (106 mg GAE/g dry plant, at 20 min) CSE yield amounted to 120 mg GAE/g dry plant, at 30 min but the extract showed similar DPPH scavenging to MAE. Maceration showed similar results to UAE.	[139]

ABTS: 2,2-azino-bis-3-ethylbenzothiazoline-6-sulfonic acid radical, ASE: accelerated solvent extraction, CA: carnosic acid, CAF: caffeic acid, CSE: conventional solvent extraction, DPPH: 1,1-diphenyl-2-picrylhydrazyl radical, MAE: microwave assisted extraction, OA: oleanolic acid, ProtCa: protocatechuic acid, RA: rosmarinic acid, SFE: supercritical fluid extraction, TPC: total phenolic content, UA: ursolic acid, UAE: ultrasound assisted extraction.

**Table 5:** Extraction yields of the major constituents of St. John’s wort reported by several investigators.

Compound	Yield (mg/g on Dry Plant Basis) Reported by Reference				
	[157]	[158] ^c^	[159] ^d^	[160] ^e^	[161] ^f^
Hyperforin	10.8–24.1 ^a^	1.5	0.7	13.0	n.d
Adhyperforin	0.4–3.2 ^a^	n.m.	n.m	2.0	n.d
Hypericin	1.5–2.6 ^b^	0.2	0.4	0.3	0.3
Pseudohypericin	0.8–1.4 ^a^	0.5	0.5	0.5	1.0
Biapigenin	7.1 ^b^		1.4	0.1	0.6
Quercetin	8.1 ^b^		1.1	3.3	2.3
Quercitrin	0.9–6.5 ^a^	0.8	1.2	1.4	2.7
Isoquercitrin	1.2–7.0 ^a^		3.3	2.6	5.2
Hyperoside	7.4–29.5 ^a^	2.8	6.2	7.3	16.3
Rutin	7.8 ^b^	3.0	0	13.0	21.4
Chlorogenic acid	1.6 ^b^		5.4	1.1	6.8

^a^: sonication with methanol at ambient temperature, ^b^: Soxhlet extraction with ethanol, ^c^: direct sonication with methanol, ^d^: repeated (24 h each) methanol extractions at dark, ^e^: ASE (120 °C, 100–150 atm) with 70% ethanol in water (solid/liquid 1/50), ^f^: Soxhlet extraction with methanol.

**Table 6:** Techniques and main results reported in literature for the extraction of phenolic compounds from St. John’s wort.

Extraction Method and Parameters	Analysis	Main Results	Reference
CSE (1 g/25 mL under stirring) Phase I: Water, ethanol 50% in water, ethanol, ethanol 50% in acetone, acetone, chloroform, hexane Phase II: ethanol in acetone 20–80%, 23–55 °C, 4.5–7.5 h	HPLC	Phase I: ethanol, ethanol 50% in acetone, and acetone were more effective for most compounds. Chloroform and hexane extracted only one compound, possibly hyperforin. Best extraction time 4–8 h. Phase II: experimental design and RSM showed optimum yield for all components 44–59% ethanol in acetone, 5.3–5.9 h, and 55 °C, except hypericin that showed maximum yield at 23 and 40 °C.	[172]
CSE (1g/30 mL, 4 °C, 60 min, under stirring and dark) Ethanol, acetonitrile, ethyl acetate, chloroform, methyl-*tert*-butylether, petroleum ether, hexane	HPLC (hyperforin)	All solvents presented close yields (3.2–2.8 mg/g dry plant), except ethanol that presented the lowest (1.9 mg/g dry plant). Hexane and petroleum ether presented the highest purity (hyperforin content) in the extracts.	[164]
SFE (311, 380, and 449 atm, 40, 50, and 60 °C).	HPLC (hyperforin)	The optimum conditions were 380 atm, 50 °C, static extraction 10 min followed by dynamic extraction 90 min at CO_2_ flow rate 1 mL/min. Extraction was not quantitative (about 60% of hyperforins were extracted). Addition of methanol did not increase yield, while decreased purity of the extract.	[163]
SFE	HPLC (hyperforin)	High extraction efficiency when CO_2_ density > 0.60 g/mL. Mild conditions (30 °C, 80 atm, density-0.64 g/mL) gave the best yield (12 mg/g dry plant) that was comparable to UAE or CSE at boiling temperature	[171]
SFE (100, 150, 200 atm, 40, 50 °C, various CO_2_ densities) UAE (methanol)	HPLC (hyperforins)	The lower the CO_2_ density (low pressure, high temperature) the lower the hyperforins yield and purity of the extracts. 200 atm and 313 K gave the best results. Hypericins were not extracted. Pretreatment with SFE (100 atm, 313 K) increased the yield of UAE extraction.	[170]
SFE (250 and 300 atm, 40 °C or 300 atm 50 °C, with or without 10% ethanol as co-solvent) Subcritical CO_2_ (70 atm, 22 °C)	HPLC (hypericin hyperforin, flavonoids)	Hyperforin was easily extracted, while hypericin and flavonoids were not extracted even with ethanol. The yield increased sharply under SFE and slower with liquid CO_2_. Liquid CO_2_ gave the highest hyperforin yield and purity of the extract. Ethanol increased hyperforin yield but decreased purity of the extract.	[169]
Soxhlet (20 g/200 mL) Ethanol, 2-propanol, ethyl acetate, hexane SFE (40 °C, 450 atm, flow rate 7 kg CO_2_/(h kg herb)	UV–vis (hypericin) HPLC (hypericin hyperforin)	Highest hypericin yield with ethanol, very low with 2-propanol and ethyl acetate, not detected in hexane and supercritical CO_2_. Hyperforin yield: supercritical CO_2_ >> Soxhlet with 2-propanol > ethanol > ethyl acetate, fully degrader in hexane due to long extraction time.	[166]
Soxhlet (5 g/100 mL) methanol 6 h successive with diethyl ether 4 h, and ethanol 6h UAE (bath, 1 g, 75 mL, 0.5 h, 2 repeated extractions) methanol successive with petroleum ether, chloroform, ethyl acetate, methanol Digestion (1 g with 100 mL hot methanol) Maceration a) 1 g/100 mL methanol, under stirring, 2 h) b) 1 g/150 mL acetone 90% in water, under stirring, 0.5 h, 2 repeated extractions)	UV–vis (hypericin) HPLC	Only Soxhlet b, and maceration b gave extracts free from chlorophyll pigments. Soxhlet b gave mainly hyperforin in diethyl ether and hypericins in ethanol. Hyperforin was favored by lower temperature (UAE a, compared to Soxhlet a) and non-polar solvents (petroleum ether in UAE b). Flavonoids were favored by higher temperature (Soxhlet a and digestion, compared to UAE a and maceration a). The best extraction procedure to obtain a representative extract with all metabolites is UAE with methanol or ethanol.	[157]
Soxhlet (5 g/150 mL methanol 24 h) UAE (5 g/100 mL methanol, 5–60 min) Direct (20 kHz, 40 or 60 W) Indirect (35 kHz) Maceration (5 g/100 mL methanol 24 h) ASE (methanol, 40 °C, 100 atm)	HPLC	Direct UAE showed the best yields for all compounds that increased with power (60 W). 20 min were efficient for all compounds, except hyperforin that needed 5 min. Yields obtained by the rest methods followed the order Soxhlet ≥ ASE ≥ indirect UAE > maceration.	[158]
Soxhlet (1 g/200 mL methanol, 1–48 h) UAE (bath, 1 g/22 mL methanol, 60 °C, 30–120 min) ASE (22–200 °C, 152 atm, 5 min heating, 5 min static time, 3-cycle extraction) Methanol, tetrahydrofuran, acetone, methylene chloride, hexane	HPLC (hyperforin was not quantitated)	Maximum yield in Soxhlet obtained at 8 h. Increase of time in UAE increased components yields. 3–6 repeated ASE were necessary for the recovery of 99% of each component. All solvents were tested at 22 °C, methanol or tetrahydrofuran were more effective for the extraction of phenolic acids and flavonoids, acetone was more effective for the extraction of the non-polar hypericins, while methylene chloride and hexane were ineffective. The effect of temperature was studied with ethanol. Yield increased with temperature up to 150 °C and decreased afterwards, except quercetin that was degraded even at 150 °C For more polar compounds yield followed the order Soxhlet (24 h) > UAE (60 °C, 2 h) > ASE (60 °C, 0.5 h).	[161]
Heating under reflux (solvent boiling point, 90 min) UAE (25 °C, 30 min) MAE (75 °C, 30 min) ASE (120 °C, 100–150 atm, 20 min) All methods with 70% methanol in water, solid/liquid 1/50	HPLC	Yield of all compounds followed the order ASE > MAE > heating under reflux > UAE. Heating under reflux at solid/liquid 1/100 presented yields comparable to ASE.	[160]
UAE (0.5 g/30 mL, bath 40 kHz) methanol in water 20–80%, HCl 0.8–2.0 M, 30–70 °C, 20–80 min	HPLC (flavonoids and phenolic acids) TPC, ABTS^+^	BBD and analysis of results indicated all parameters significant. Optimization was based on quercetin yield that increased with methanol concentration and temperature, while it was not affected by HCl concentration at higher temperature. Cyanidin, kaempferol, and protocatechuic acid were also found in the extract.	[176]
CSE (1 g/50 mL under stirring) Water Glycerol in water 10% 50–70 °C, 5–95 min	HPLC (phenolic compounds) TPC, FRAP	Aqueous glycerol (10%) increased the extraction rate, compared to water, the phenolic compound yield (90 mg GAE/g dry herb, versus 78 mg GAE/g dry herb for water), and the ferric reducing power (by 9%). Phenolic acids, quercetin glycosides, and catechin were the major extracted components, while hypericin was detected. CCD and RSM revealed 70 °C and 69 min as optimum conditions.	[177]

ABTS: 2,2-azino-bis-3-ethylbenzothiazoline-6-sulfonic acid radical, ASE: accelerated solvent extraction, CSE: conventional solvent extraction, FRAP: ferric reducing antioxidant power, MAE: microwave assisted extraction, SFE: supercritical fluid extraction, TPC: total phenolic content, UAE: ultrasound assisted extraction.

**Table 7:** The extraction parameters of *Crocus sativus* stigmas, as recorder from the extensive review of literature.

Solvent	Method	Measured Parameters	Main Results	Reference
Water (solid/liquid 1/2000, *w/v*)	CSE with magnetic stirring at 1000 rpm for 1 h under dark (ISO 3632-2:2010)	UV–vis spectrophotometry	picrocrocin, safranal and crocins are expressed as direct reading of the absorbances produced by the 1:10 dilution of the extract at 257, 330 and 440 nm	[192]
Methanol, ethanol, propanol, acetone, ethyl acetate and petroleum ether (solid/liquid 1/50, *w/v*)	Soxhlet Cold percolation	UV–vis spectrophotometry, HPLC-UV	Soxhlet (overnight): acetone recovered picrocrocin in the highest yield, while methanol was more effective for the extraction of safranal and crocins. Cold percolation (overnight): the safranal content of oleoresin remain more intact in this method	[193]
Ethanol–water mixtures (solid/liquid 1/10–1/40, *w/v*)	CSE (agitation at 200 rpm for up to 60 min, at 25 °C, and protected from light	HPLC-DAD, TPC	Optimization for crocins: ethanol 50% (*v/v*), temperature 25 °C, solid/solvent 1/20; recovery 77% of theoretical	[194]
Distilled water (DW), ethanol/DW, methanol/DW, propylene glycol/DW, heptane/DW and hexane/DW (solid/liquid 1/30, *w/v*)	Maceration (72 h, 25 °C)	UV–vis spectrophotometry	Ethanol–water was the most efficient solvent for the extraction of crocin and safranal, while methanol–water was the most efficient for picrocrocin	[195]
Ethanol in water 50% (solid/liquid 1/10, 1/20 *w/v*)	MAE: 200 W (under magnetic stirring, 50 °C, 18 min) UAE: (33 KHz, room temperature, 30 min)	ABTS, DPPH, FRAP, TPC	The MAE method was more effective compared to the UAE method, with six fold higher yield.	[196]
Methanol in water 50% (solid/liquid 1/180–1/1800, *w/v*)	UAE. Amplitude setting range, 10–100 in 1% increments; frequency, 20 kHz and output, 70 W; 100% amplitude; temperature: 15 ± 0.5 °C	HPLC-DAD, Optical Microscopy	Optimal conditions for crocins recovery: solid/liquid 1/180 *w/v*; sonication time: 29 min; yield 627 mg/g saffron	[197]
Ethanol in water 50% (solid/liquid 1/20, *w/v*)	UAE. frequency 25 kHz; power: 100 W; sonication time 1–10 min; temperature 25 °C	UV–vis spectrophotometry	Optimal time to extract crocin, picrocrocin and safranal was 10 min. The yield was higher than with maceration for 72 h.	[198]
Water (solid/liquid 1/100, *w/v*)	Hydration of the ground material for 2 h at 4 °C, and then application of high hydrostatic pressure	UV–vis spectrophotometry (ISO method), HPLC-DAD	Optimal conditions for maximum extraction of safranal, picrocrocin and crocin: 5800 atm and 50 °C. Total yield of crocins more than 250 mg/g	[199]
CO_2_	SFE 200 atm, 100 °C	Safranal GC-FID, HPLC-DAD	0.476 g/mL fluid density (200 atm and 100 °C); total extraction of safranal within 30 min	[200]
Supercritical CO_2_ (solid/S. Fluid 1/3755, *w/v*)	SFE Extraction between 30 and 190 min	GC-MS	Optimal conditions: temperature 44.9 °C; pressure 349 atm; extraction time 150.2 min; CO_2_ flow rate 10.1 L h^−1^; yield 10.94 mg/g from the non-polar fraction	[201]
CO_2_ and CO_2_-methanol (solid/S. Fluid 1/73, *w/v*)	SFE	HPLC-UV/vis detector	Optimal recovery of crocin (32.67% *w/w*): 44 °C, 193 atm, 1.0 cm^3^/min, 110 min Safranal (recovery 91.8% *w/w*): 92 °C, 213 atm, 0.9 cm^3^ min, 122 min	[202]
CO_2_ (solid/S. Fluid 1/1440; solid/liquid methanol or water as modifier, 1/96 /V)	SFE; duration 240 min; CO_2_ pump flow rate 3 mL/min; modifier flow rate of 0.2 mL/min.	HPLC-UV/vis detector	Crocin was optimally extracted at 80 °C and 300 atm using water as a modifier. Optimal conditions for safranal: 80 °C and 400 atm using methanol as a modifier	[203]

ABTS: 2,2-azino-bis-3-ethylbenzothiazoline-6-sulfonic acid radical, CSE: conventional solvent extraction, DPPH: 1,1-diphenyl-2-picrylhydrazyl radical, FRAP: ferric reducing antioxidant power, MAE: microwave assisted extraction, SFE: supercritical fluid extraction, TPC: total phenolic content, UAE: ultrasound assisted extraction.

**Table 8:** The extraction parameters of *Crocus sativus* petals, as obtained from the review of literature.

Solvent	Method	Measured Parameters	Main Results	Reference
Water (solid/liquid 1/36, *w/v*)	ASE (SWE)	UV–vis spectrophotometry TPC, TFC, DPPH, FRAP	Optimum conditions: 159 °C, 54 min TPC yield 16.2 mg/g, and flavonols yield 2.39 mg/g.	[208]
Ethanol–water (solid/liquid 1/20, *w/v*)	Maceration	UV–vis spectrophotometry TAC	ethanol concentration in water 25%, extraction temperature 25.8 °C, duration of extraction 24 h; total monomeric anthocyanins yield 32 mg/g	[209]
CO_2_ (solid/liquid 1/30, *w/v*; 5% *v/v* ethanol)	SFE	UV–vis spectrophotometry TPC, TFC, TAC, DPPH, FRAP	62 °C, 47 min extraction time and pressure 164 atm; TPC yield 14.7 mg/g, TFC 1.8 mg/g, TAC 1 mg/g	[210]
Ethanol–water 59:41 (solid/liquid 1/20 (CSE), 1/30 (UAE), 1/50 (MAE), *w/v*)	CSE, UAE, MAE	UV–vis spectrophotometry TPC, TAC, DPPH	Maceration: 66 °C for 15 min Yields: TPC 45 mg/g, TAC 4.6 mg/g UAE: 66 °C for 2 min Yields: TPC 47 mg/g, TAC 5.3 mg/g MAE: 66 °C for 2 min Yields: TPC 43 mg/g, TAC 5.2 mg/g	[211]
Ethanol–water 50:50, 25:75 acidified with HCL 0.1 N up to pH = 2 (solid/liquid 1/77.5, *w/v*)	MAE	UV–vis spectrophotometry TAC	MAE: temperature 48 °C, power 360 W, extraction time 9.3 min; TAC yield 101 mg/g	[212]
Water (0.3% *w/v* NaCl) (solid/liquid 1/20)	CHWE, OHAE, UAE, MAE	UV–vis spectrophotometry TPC, TFC, TAC, DPPH LC-MS	CHWE: agitation, 66 °C for 104 min; (dry herb) TPC 7.21 mg/g TFC 1.01 mg/g, TAC 1.89 mg/g OHAE: 45 min-225 V; (dry herb) TPC 9.28 mg/g, TFC 1.48 mg/g, TAC 2.38 mg/g MAE: 4.25 min-500 W; (dry herb) TPC 8.69 mg/g, TFC 1.15 mg/g, TAC 2.06 mg/g UAE: 40.61 min-135.3 W; (dry herb) TPC 8.63 mg/g, TFC 1.30 mg/g, TAC 2.05 mg/g	[213]
Water, Methanol–water (solid/liquid 1/50)	maceration, UAE	UV–vis spectrophotometry TPC, TAC, FRAP, ABTS, DPPH HPLC-DAD	Maceration with water under stirring (1000 rpm) under dark for 30 min, at 21 °C: TPC 11.4 mg/g, TAC 3.45 mg/g UAE with water at 23 kHz for 15 min: TPC 11.5 mg/g, TAC 4.13 mg/g	[207]

ABTS: 2,2-azino-bis-3-ethylbenzothiazoline-6-sulfonic acid radical, ASE: accelerated solvent extraction, CHWE: conventional hot water extraction, CSE: conventional solvent extraction, DPPH: 1,1-diphenyl-2-picrylhydrazyl radical, FRAP: ferric reducing antioxidant power, MAE: microwave assisted extraction, OHAE: Ohmic heating assisted extraction, SFE: supercritical fluid extraction, SWE: subcritical water extraction, TAC: Total Anthocyanin content, TPC: total phenolic content, TFC: Total flavonoids content, UAE: ultrasound assisted extraction.

## Supplementary Materials

The following are available online. Table S1: Identified phenolic compounds in Lamiaceae herbs extracts reported in literature references.

## Abbreviations

ABTS: 2,2-azino-bis-3-ethylbenzothiazoline-6-sulfonic acid radical, ASE: accelerated solvent extraction, BBD: Box–Behnken design, CA: carnosic acid, CAF: caffeic acid, CAR: carvacrol, CCD: Central composite design, CHWE: conventional hot water extraction, COH: carnosol, CSE: conventional solvent extraction DPPH: 1,1-diphenyl-2-picrylhydrazyl radical, EO: essential oil, HPLC: high pressure liquid chromatography, FRAP: ferric reducing antioxidant power, GAE: gallic acid equivalents, MAE: microwave assisted extraction, OA: oleanolic acid, OHAE: Ohmic heating assisted extraction, ORAC: oxygen radical absorbance capacity, PLE: pressurized liquid extraction, PHWE: pressurized hot water extraction, ProtCa: protocatechuic acid, PWE: pressurized water extraction, RA: rosmarinic acid, RSM: response surface methodology, SFE: supercritical fluid extraction, SFME: solid free microwave extraction, SWE: subcritical water extraction, TEAC: trolox equivalent antioxidant capacity, TPC: total phenolic content, UA: ursolic acid, UAE: ultrasound assisted extraction, WEPO: pressurized water extraction with particle on-line formation.
