# Supplementary material for: Six Common Herbs with Distinctive Bioactive, Antioxidant Components. A Review of Their Separation Techniques
Source: Molecules. 2021 May 14;26(10):2920. doi: 10.3390/molecules26102920 (PMC8157015; doi:10.3390/molecules26102920)
Supplement: Supplementary file 1 [file molecules-26-02920-s001.zip › molecules-1214159-supplementary.pdf]

Table S1. Identified phenolic compounds in Lamiaceae herbs extracts reported in literature references.

Compound	Rosemary	Oregano	Pink savory	Lemon balm
			Reference	
**Hydroxybenzoic Acids**				
4-hydroxybenzoic acid	17,71,79,106	71,66,79	79	79,106,129
Protocatechuic acid	71,79,106	69,71,79	79	79,106,119,126,127,128
Gentisic acid	106			106,128
Vanillic acid	7179,106	71,79	79	79
Gallic acid	30,71,79	71,79	79	79,116,118,128,129
Ellagic acid				116
Syringic acid	71,79,106	71,79	79	79,118
**Hydroxycinnamic Acids**				
trans-Cinnamic acid	79	79	79	
p-Coumaric acid	17,71,79,106	66,71,79	79	66,128,129
Ferulic acid	71,79,106	66,71,79	79	79,118
Sinapic acid	79			
Caffeic acid/derivatives	8,17,27,37,71,79,106	64,66,71,73,75,79	2,79,99,101,108,109	66,79,106,113,116,118,119,125,126,127,128,129
3-O-Caffeoylquinic acid		69		
5-O-Caffeoylquinic acid		69		
Caftaric acid				126,127,128
Chicoric acid				128
Chlorogenic acid	17,71,79	66,71,79	79	79,116,118,129
Dicaffeoylquinic acid	71	71		
Lithospermic acids		64,69,73	99,101,108,109	124,126,127
Rosmarinic acid/derivatives	17,19,20,21,27,37,71,79,106	64,65,66,69,71,73,75,79	2,79,99,101,107, 108,109	66,79,106,113,117,118,119,124,125,129
Salvianolic acids	21	64	99,101,109	117,124,126
Quinic acid				126
**Flavonoids**				
Acacetin	8,71	71		
Apigenin	8,17,19,71	71,72,73,75,79	79,99,100,101,107,108,109	79
Apigenin-7-*O*-glucoside	17,19,71	71	101,109	126
Apigenin glycoside			99	
Apigenin diglycoside			108,109	
Apigenin glucuronide		69		
Methyl apigenin O-glucuronide		69		
Apigenin-7-*O*-rutinoside	17,71	66,69,71		
Apigenin-rhamnoglucoside	27			
Apigenin 6,8-di-C-glycoside		64,69	99,100	
methyl apigenin	71	71		
6-OH apigenin 7,3′-dimethyl ether			99	

Aromadendrin		72,88	100,101	
Catechin	79	79	79	79,116
Chryseriol		73		
Cirsilineol			100	
8-methoxy cirsilineol			100	
Cirsimaritin	8,17,19,20,27,37,71	71	100	
Cirsimaritin glucoside	17,27			
Cosmoside		73		
Diosmetin	19	73		
Diosmetin-rhamnoglucoside	27			
Epicatechin			79	79,116
Epigallocatechin		79		79
Eriodictyol	17	64,69,72,73,88	99,100,101,108,109	
Gallocatechin	19,20,37			
Genkwanin	8,17,19,20,21,27,37	72	100	
Hesperetin	17			
Hesperidin	17, 19,20,27			
Hispidulin	19,21			
Hispidulin-*7*-*O*-glucoside	27			
Hispidulin-rutinoside	17			
Homoplantaginin	17,19,20,37			
Isoscutellarein	21			
Isoscutellarein-*7*-*O*-glucoside	27			
Isorhamnetin	17			
Isorhamnetin-3-*O*-hexoside	17			
Isorhamnetin-rutinoside	17			
Isosakuranetin	17			
Kaempferol/derivatives		69,79	79	79
Kaempferol diglycoside				66
Ladanein	21	72,	100	
Luteolin	17,19,71,79	71,73,79	79,99,101,107,109	79,117,126
Luteolin-3′-acetyl-*O*-glucuronide	17,19,20			
Luteolin-3′-*O*-glucuronide	20,71	71	107	124,125
Luteolin-hexoside	17			
Luteolin-7-*O*-glucuronide	17, 19,27	69		
Luteolin-*7*-*O*-glucoside	71	69,71,75	107	125,126
Luteolin-7-*O*-rutinoside	17,19	71	101	
Luteolin 7-methyl ether			100	
luteolin 7,4′-di-O-glucuronide			101,108,109	
6-OH luteolin 7,3′-dimethyl ether			99,100,101,108,109	
6-OH luteolin 7,3′,4′-trimethyl ether			99,100,101,108,109	
Myricetin	79		79	79,129
Myricetin 3-*O*-glucoside		69		
Naringenin	79	69,72,75,79,88	79,99,100,101,108,109	

Nepitrin	19,20,21			
Pebrellin		72		
Phloridzin	71	71		
Rutin	79	71,79	79	116,129
Salvigenin	19,21			
Scutellarein	8,19,37			
6,7-Dimethoxy scutellarein	71	71		
Sorbifolin		72		
Taxifolin		69,72,88	100	
4'-Methoxy tectochrisin	21,27			
Quercetin/ derivatives	79	69,71,73,88	99,101,107,109	79,116,129
Quercetin glucoside			107	66
Quercetin-3-*O*-hexoside	71	71,		
**Triterpenic Acids**				
Ursolic acid	18,20	65,66		113,114,125,139
Oleanolic acid	18	65		113,114,125,139
**Phenolic diterpenes**				
Carnosic acid/derivatives	17,19,20,21,27,71	71		125,139
Carnosol/derivatives	17,19,20,21,27,71	71		
Rosmanol/derivatives	8,17,19,20,27			
